# Alcohol use disorder–associated gene *FNDC4* alters glutamatergic and GABAergic neurogenesis in neural organoids

**DOI:** 10.1172/JCI193204

**Published:** 2026-01-08

**Authors:** Xiujuan Zhu, August J. John, Sooan Kim, Li Wang, Enci Ding, Jing Zheng, Ateka Saleh, Irene Marín-Goñi, Abedalrahman Jomaa, Huanyao Gao, Meijie Wang, Ching Man Wai, Irene Moon, Cindy Chen, Alireza Agahi, Brandon J. Coombes, Tony M. Kerr, Nobuyoshi Suto, Liewei Wang, Mark A. Frye, Joanna M. Biernacka, Victor M. Karpyak, Hu Li, Richard M. Weinshilboum, Duan Liu

**Affiliations:** 1 Department of Molecular Pharmacology and Experimental Therapeutics, Mayo Clinic, Rochester, Minnesota, USA.; 2 Department of Biochemistry and Molecular Genetics, Feinberg School of Medicine, Northwestern University, Chicago, Illinois, USA.; 3 Department of Quantitative Health Sciences and; 4 Department of Psychiatry and Psychology, Mayo Clinic, Rochester, Minnesota, USA.

**Keywords:** Genetics, Neuroscience, Addiction, Molecular genetics, Pharmacogenetics

## Abstract

Large-cohort GWAS for alcohol use disorder (AUD) drug treatment outcomes and AUD risk have repeatedly identified genetic loci that are splicing quantitative trait loci for the *fibronectin III domain containing 4* (*FNDC4*) gene in the brain. However, *FNDC4* function in the brain and how it might contribute to AUD pathophysiology remain unclear. In the present study, we characterized GWAS loci–associated *FNDC4* splice isoforms and demonstrated that *FNDC4* alternative splicing results in loss of function for FNDC4. We also investigated *FNDC4* function using CRISPR/Cas9 editing and the creation of human induced pluripotent stem cell–derived (iPSC-derived) neural organoids joined with single-nucleus RNA sequencing, a series of studies that showed that *FNDC4* KO resulted in a striking shift in the relative proportions of glutamatergic and GABAergic neurons in iPSC-derived forebrain organoids as well as changes in their electrical activity. We further explored a potential mechanism(s) of *FNDC4*-dependent neurogenesis, and the results suggested a role for FNDC4 in mediating neural cell surface interactions. In summary, this series of experiments indicates that *FNDC4* plays a role in regulating cerebral cortical neurogenesis in the brain. This regulation may contribute to the response to AUD pharmacotherapy as well as the effects of alcohol on the brain.

## Abstract 2

The study reveals that the *FNDC4* gene affects alcohol use disorder risk and drug treatment outcomes through its function in neurogenesis.

## Introduction

Alcohol use disorder (AUD) is the most common addictive disorder and a leading cause of disease burden worldwide (1, 2). Patients with AUD experience intense alcohol craving that drives increased alcohol ingestion despite harmful health consequences (1, 2). It is believed that AUD may develop as a result of disrupted homeostasis of neurotransmission after exposure to alcohol, which can influence multiple brain neurotransmitter systems (3). However, not every subject who drinks alcohol will develop AUD, indicating that susceptibility to this disorder is multifactorial. Genetics clearly contributes to AUD risk (4). Recently, large-cohort human GWAS of AUD and AUD-related phenotypes have identified a large number of genetic loci (5–7), results which have substantially advanced our understanding of the genetic etiology of AUD. Some of the GWAS-identified loci appeared to be aligned with known mechanisms of AUD pathophysiology, e.g*.*, alcohol metabolism (*ADH1B* and *ADH1C*) and neurotransmission (*DRD2, GABRA4,* *OPRM1,* and *CACNA1C*). However, like many GWAS, the majority of AUD GWAS-identified loci mapped to noncoding regions, and their function in AUD needs to be investigated further (4).

Only 3 medications are currently approved by the FDA for use in the treatment of AUD, with acamprosate and naltrexone being the most widely prescribed (2, 8). Both drugs are believed to target mechanisms of neurotransmission. However, response to treatment with these medications is variable, with only approximately 50% of patients with AUD achieving substantial positive treatment outcomes (8, 9). To better understand the contribution of genetic factors to individual variation in response to AUD pharmacotherapy, we performed a GWAS for treatment outcomes that included over 1,000 patients with AUD who received acamprosate and/or naltrexone therapy (9). The GWAS identified a genome-wide significant SNP locus that colocalized with a splicing quantitative trait locus (sQTL) for the *fibronectin III domain containing 4* (*FNDC4*) gene in multiple human brain regions (10). We also noted an independent SNP locus that is also an sQTL for *FNDC4* in the human brain and has been repeatedly associated with AUD risk and alcohol consumption in large-cohort GWAS (5–7). The fact that brain *FNDC4* sQTL SNPs are significantly associated with both AUD risk and AUD drug treatment outcomes strongly suggests a possible role for brain FNDC4 in AUD pathophysiology. However, despite the fact that *FNDC4* is highly expressed in the brain (10), its function in the CNS and how that function might contribute to AUD pathophysiology remain unclear.

In the present study, we characterized the molecular function of *FNDC4* in the CNS with the goal of understanding its possible role in AUD pathophysiology and its contribution to variation in AUD drug treatment response. We began with functional annotation and characterization of the GWAS SNP–associated *FNDC4* RNA splice isoforms, studies which suggested that the GWAS SNPs might be associated with FNDC4 decrease or loss of function in the brain. Next, we studied the *FNDC4* function in human induced pluripotent stem cell–derived (iPSC-derived) neural progenitor cells and dorsal forebrain organoids using a series of assays including co-IP, CRISPR/Cas9, single-nucleus RNA sequencing (snRNA-seq), and microelectrode arrays (MEAs). This series of experiments suggested that FNDC4 may play a role in regulating glutamatergic and GABAergic neurogenesis though cell surface interactions, at least at certain early stages of neurodevelopment, a function that may contribute to the effect of alcohol on the brain and clinical response to AUD drug therapy.

## Results

### Potential association of brain FNDC4 alternative splicing with AUD phenotypes.

**Figure 1:**
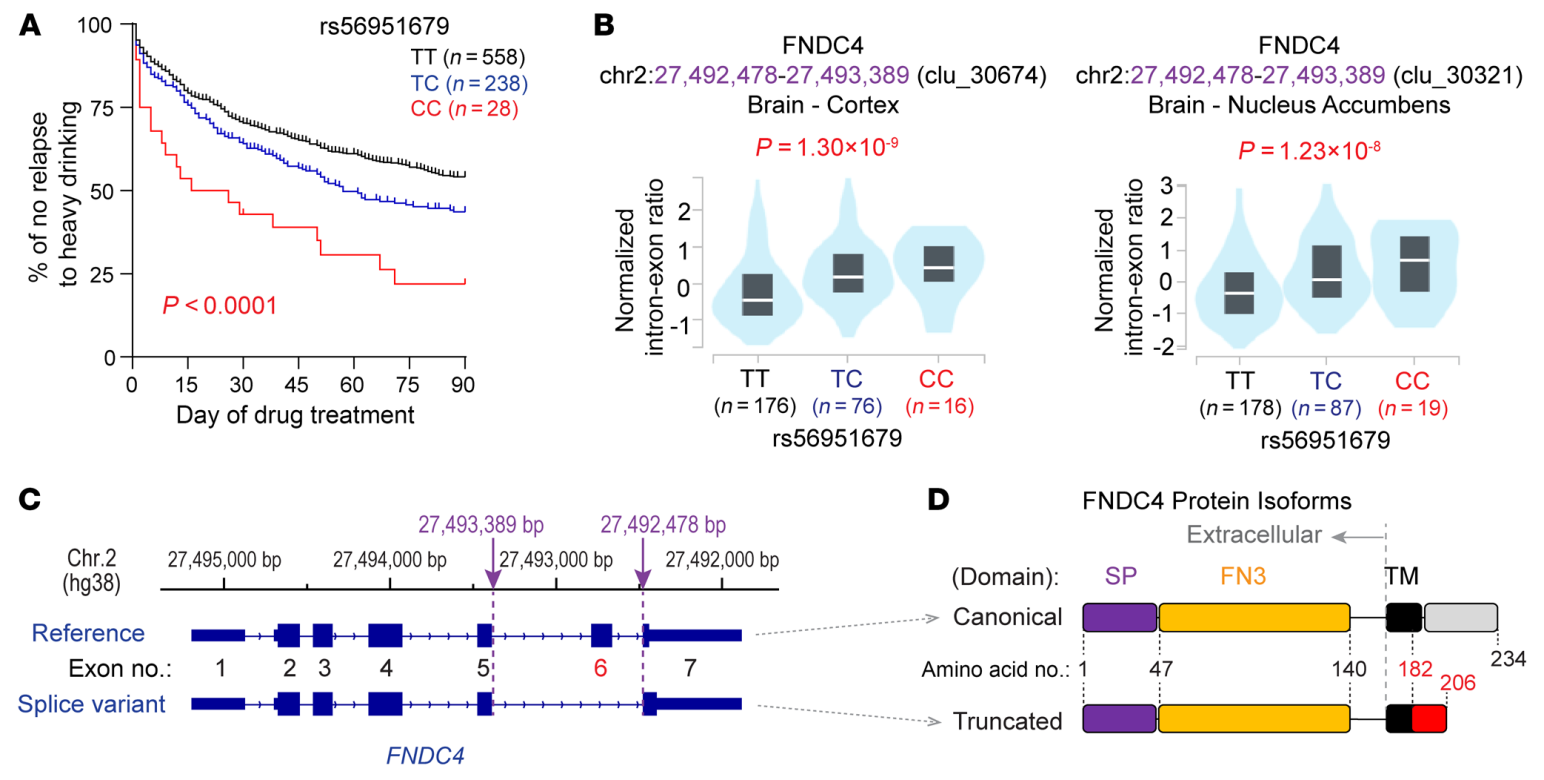
AUD drug treatment response and *FNDC4* alternative splicing in the brain. (**A**) Kaplan-Meier plot showing the percentage of patients with AUD who did not relapse to heavy alcohol drinking during 90 days of pharmacotherapy. A smaller percentage of patients who carried the rs56951679 SNP variant allele C remained abstinent from heavy alcohol drinking, suggesting that worse drug treatment response is seen in patients carrying the rs56951679 SNP C allele. The *P* value was calculated using the log-rank (Mantel-Cox) test. The Kaplan-Meier plot was generated based on our published GWAS data (9). (**B**) The rs56951679 SNP is an sQTL for the *FNDC4* gene in many human brain regions. The SNP genotype was associated with FNDC4 RNA splicing in brain cortex (left) and nucleus accumbens (right) based on the RNA-seq data generated by GTEx (v10) (10). The variant allele C was associated with an increased level of intron excision on chromosome 2 (chr.2): 27,492,478-27,493,389 (hg38). (**C**) Depiction of the rs56951679 SNP-associated alternative splice sites and FNDC4 RNA splice isoforms. The physical position of the *FNDC4* gene on chr.2 based on human genome assembly hg38 with sQTL splice sites highlighted in purple (top); the reference FNDC4 mRNA with 7 exons (middle) and FNDC4 splice variant with exon 6 excision (bottom) are numbered below. FNDC4 transcripts were annotated by RefSeq. (**D**) Depiction of the FNDC4 protein encoded by the reference (canonical) and spliced (truncated) FNDC4 RNA isoforms. The canonical FNDC4 protein includes a signal peptide (SP), a FN3 domain, a TM domain, and a cytosolic C-terminus. The truncated FNDC4 protein displays a frameshift after amino acid 182 that disrupts the TM domain.

Our previous GWAS for AUD drug treatment outcomes identified a genome-wide significant SNP signal (top SNP, rs56951679, T>C; *P* = 1.6 × 10^−8^) that was associated with “time until relapse to heavy alcohol drinking” after 90 days of acamprosate and/or naltrexone therapy (9). The minor C allele was associated with worse treatment outcomes (Figure 1A). The rs56951679 SNP locus colocalized with an *FNDC4* sQTL in multiple brain regions, including cortex, nucleus accumbens, and 3 other brain regions based on data from the Genotype-Tissue Expression (GTEx) project (10) (Figure 1B and Supplemental Figure 1; supplemental material available online with this article; https://doi.org/10.1172/JCI193204DS1). The minor allele, C, was associated with increased intron/exon ratio at splice sites (chromosome 2: 27,493,389-27,472,478 bp) that excise exon 6 of the FNDC4 reference mRNA (Figure 1C), indicating an increased level of the FNDC4 splice variant mRNA in the brains of individuals who carry the C allele. In addition, large-cohort GWAS for AUD risk and alcohol consumption have repeatedly identified a different SNP locus (index SNP, rs1260326, C>T) (5–7) that also colocalized with an sQTL for *FNDC4* in the human brain (10) (Supplemental Figure 2). The rs1260326 SNP common allele C that was associated with increased AUD risk in large-cohort GWAS (5–7) was also associated with increased intron/exon ratio at splice sites in which exon 6 had been deleted (Supplemental Figure 2).

Both our GWAS for AUD drug treatment response (9) and the GWAS for AUD risk (5–7) identified SNP loci that colocalized with *FNDC4* sQTLs in brain tissue, results that suggested a potential correlation between brain FNDC4 splicing and AUD clinical phenotypes. Specifically, increased levels of the FNDC4 splice variant mRNA in the brain were associated with worse AUD drug treatment outcomes as well as elevated AUD risk. The FNDC4 splice variant mRNA, with loss of the exon 6, resulted in a truncated protein, a change that could potentially affect FNDC4 subcellular localization. Specifically, as encoded by reference mRNA, the canonical FNDC4 protein included a signal peptide, a fibronectin type III (FN3) domain, a transmembrane (TM) domain, and a cytosolic C-terminal domain (Figure 1D). As a result, it was predicted to be a single-pass type I TM protein with 3 putative N-linked glycosylation sites (11). However, the truncated protein, as encoded by the FNDC4 splice variant, had a change in the amino acid sequence, which interrupted part of the TM domain (Figure 1D). This change could potentially affect FNDC4 cell membrane localization.

Although the colocalization of AUD GWAS loci with *FNDC4* sQTLs in the brain does not establish a causal relationship between *FNDC4* and AUD phenotypes, these human population–based multi-omics data strongly support a possible role for variation in brain expression of FNDC4 in alcohol-related phenotypes. Therefore, we set out to characterize *FNDC4* function to better understand a biological mechanism(s) that might contribute to AUD-related phenotypes.

### FNDC4 splice variant encodes a truncated protein with loss of cell membrane localization and glycosylation.

**Figure 2:**
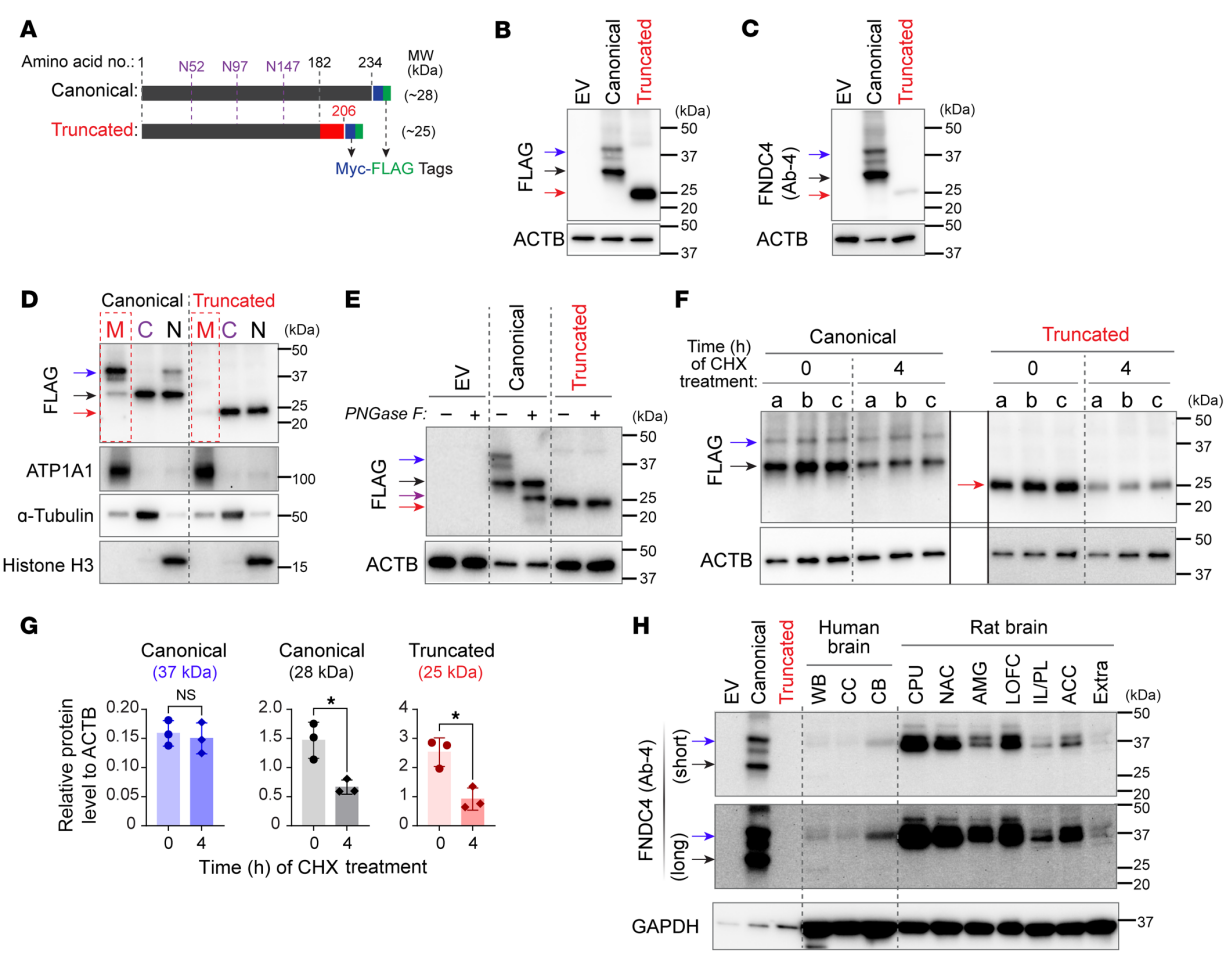
FNDC4 protein isoform characterization. (**A**) Depiction of the cDNA-overexpressed Myc-FLAG–tagged FNDC4 protein isoforms. Three putative N-linked glycosylation sites are labeled purple. Their predicted MWs are also listed. (**B**) Overexpressed FNDC4 proteins detected by anti-FLAG antibody. Compared with empty vector (EV), canonical FNDC4 showed multiple bands (blue and black arrows), while the truncated protein showed only 1 major band (red arrow). ACTB was blotted as a loading control. (**C**) The same FNDC4 overexpression samples were detected by an anti-FNDC4 antibody (Ab-4). (**D**) Overexpressed FNDC4 proteins in subcellular fractions of the membrane (M), cytoplasm (C), and nucleus (N). The larger bands (blue arrow) of canonical FNDC4 mapped mainly to membrane, while the smaller band (black arrow) mapped mainly to cytoplasm and nucleus. Truncated FNDC4 (red arrow) was located mainly in cytoplasm and nucleus but not membrane. ATP1A1, α-tubulin, and histoneH3 were blotted as markers for the membrane, cytoplasm, and nucleus, respectively. (**E**) Overexpressed FNDC4 proteins were treated with the PNGaseF. The treatment (+) removed the larger bands (blue arrow) of canonical FNDC4; simultaneously, a smaller band emerged (purple arrow). The truncated FNDC4 band was not affected by PNGaseF. (**F**) Overexpressed FNDC4 proteins in HEK293T cells before and after 4 hours of treatment with cycloheximide (CHX), which inhibits protein synthesis. Lanes a–c are biological triplicates (*n* = 3/group). (**G**) Relative protein levels of overexpressed FNDC4 to ACTB. Protein levels were quantified based on band intensity shown in **F**. *P < 0.05, unpaired, 2-tailed Student’s *t* test. Data are shown as the mean ± SD. (**H**) FNDC4 in different regions of human and rat brains. Whole-brain (WB), cerebral cortex (CC), cerebellum (CB), caudate putamen (CPU), nucleus accumbens (NAC), amygdala (AMG), lateral orbitofrontal cortex (LOFC), infralimbic/prelimbic cortices (IL/PL), anterior cingulate cortex (ACC), and extra forebrain tissues containing medial orbitofrontal cortex and motor cortex (Extra). GAPDH was blotted as an internal control.

Our functional studies began with characterizing the truncated protein encoded by the GWAS SNP–associated FNDC4 splice variant. As a first step to determine whether the truncated protein might or might not translocate to the cell membrane, we overexpressed both the canonical and truncated FNDC4 proteins in HEK293T cells with Myc- and FLAG-tags fused to their C-termini (Figure 2A). MWs for the overexpressed Myc-FLAG–fused FNDC4 proteins were approximately 3 kD larger than the predicted MWs for the native proteins, which were 25.2 kD (canonical) and 22.7 kD (truncated). We validated overexpression by Western blot using either FLAG (Figure 2B) or MYC antibodies (Supplemental Figure 3A). We observed multiple bands for the overexpressed canonical FNDC4 protein. One of those bands matched its predicted MW of 28 kD (Figure 2B, black arrow), while other bands had higher MWs close to 37 kD (Figure 2B, blue arrow). By contrast, only 1 major band with a MW that matched its predicted size was observed for the truncated protein (Figure 2B, red arrow). Using the same overexpressed FNDC4 protein samples as positive controls, we tested multiple commercially available anti-FNDC4 antibodies (Supplemental Figure 3, B and C, and Supplemental Table 1). The most efficient anti-FNDC4 antibody (Ab-4; Figure 2C) was then used to study endogenous FNDC4 protein in brain tissue.

We next determined the subcellular localization of both the canonical and truncated FNDC4 proteins by Western blot analysis after subcellular fractionation of cell membrane, cytoplasm, and nucleus of HEK293T cells that overexpressed FNDC4 protein. For the canonical FNDC4 protein, we found that the approximately 37 kD bands (Figure 2D, blue arrow) were present predominantly in the cell membrane and that the approximately 28 kD band (Figure 2D, black arrow) was present in the cytoplasm and nucleus. By contrast, the truncated FNDC4 protein was present mainly in the cytoplasm and nucleus but not in the cell membrane (Figure 2D, red arrow). These results demonstrated that the canonical FNDC4 protein could translocate to the cell membrane but that the truncated protein, encoded by the GWAS SNP–associated FNDC4 splice variant, could not efficiently translocate to the cell membrane.

FNDC4 has been studied in peripheral tissues, but the Western blot results for those studies are contradictory, with 2 studies reporting multiple bands (12, 13) and 1 reporting only a single band (14). Since we had observed multiple bands for the canonical FNDC4 protein and, most importantly, since those bands were distributed at different subcellular localizations (Figure 2D), we next investigated what those multiple bands might represent. We found that the cell membrane–enriched bands (Figure 2E, blue arrow) represented glycosylated canonical FNDC4 protein because those bands disappeared, and a smaller band appeared (Figure 2E, purple arrow), after the protein sample had been treated with peptide:*N*-glycosidase F (PNGaseF), an endoglycosidase that can cleave N-linked oligosaccharides from glycoproteins (15). By contrast, the truncated FNDC4 protein band was not affected by PNGaseF exposure (Figure 2E, red arrow), suggesting that, unlike the canonical protein, the truncated protein was not glycosylated. This result was consistent with bioinformatic predictions that FNDC4 had 3 putative N-linked glycosylation sites (Figure 2A) and the fact that glycoproteins often show higher MWs in Western blots than their predicted MWs (16, 17). The MW of the newly appearing band (Figure 2E, purple arrow) was smaller than that of the cytoplasm-enriched band (Figure 2E, black arrow), which likely was due to both deglycosylation by PNGaseF and cleavage of the signal peptide, which often occurs when a newly synthesized protein is translocated to the membrane (18).

We also observed that the unglycosylated canonical and truncated FNDC4 proteins (Figure 2F, black and red arrows) were less stable than the glycosylated FNDC4 protein (Figure 2F, blue arrow). Specifically, when HEK293T cells overexpressing FNDC4 protein were treated with cycloheximide (which inhibits protein synthesis) for 4 hours, significant decreases in unglycosylated canonical and truncated FNDC4 proteins were observed, but that was not the case for glycosylated FNDC4 (Figure 2, F and G).

In summary, this series of experiments demonstrated that the GWAS SNP–associated FNDC4 splice variant encoded a truncated protein that could not be efficiently glycosylated or translocated to the cell membrane, resulting in an unstable and rapidly degraded protein in the cytoplasm when compared with the glycosylated canonical protein. This result suggested that the GWAS SNP–associated FNDC4 splice variant in the brain might have been associated with AUD-related phenotypes because it encoded an unstable truncated protein that compromised FNDC4 function in the brain. These experiments characterized FNDC4 protein isoforms in detail, a crucial initial step for the study of endogenous FNDC4 in the brain, as described below.

### FNDC4 is primarily a TM glycoprotein in the brain.

Before studying FNDC4 function using CNS models, we attempted to detect endogenous FNDC4 protein isoforms in the human brain since that information would help us understand its function in the brain. We used a validated FNDC4 antibody (Ab-4 in Figure 2C) to detect FNDC4 proteins in commercially available human brain protein lysates by Western blot. The experiments showed major bands with MWs that matched that of the glycosylated canonical FNDC4 protein present in human whole-brain, cerebral cortex, and cerebellum lysates (Supplemental Figure 3D).

To further characterize endogenous FNDC4 protein isoforms in the brain, we harvested both cortical and subcortical regions of rat brains and snap-froze them to obtain protein lysates. This step made it possible to generate protein lysates from fresh brain tissue — a source that we could not obtain from human subjects for obvious reasons. FNDC4 protein sequences are highly conserved across mammalian species. For example, human and rat FNDC4 protein sequences are almost identical, with less than 5% differences found in the signal peptide (Supplemental Figure 4), a domain that is often cleaved after proteins are translocated to the cell membrane (18). Therefore, we anticipated that the human FNDC4 antibody would also detect rat FNDC4 in Western blot. In fact, we detected endogenous FNDC4 bands in multiple rat brain regions (Figure 2H). These FNDC4 bands were more intense than those detected in human brain lysates, observations that might be due to the method used to prepare and store protein lysates. Importantly, the MWs of FNDC4 bands in both human and rat brain lysates were similar (~37 kD), and they matched well with the glycosylated membrane-enriched canonical FNDC4 protein (Figure 2H, blue arrows). By contrast, the unglycosylated cytoplasm-enriched canonical FNDC4 protein (Figure 2H, black arrows) was barely detectable in either human or rat brain lysates, perhaps due to a rapid degradation.

Of note, although this FNDC4 antibody (Ab-4) detected overexpressed truncated FNDC4, its efficiency in detecting truncated FNDC4 was much lower than in detecting canonical FNDC4 (Figure 2C). It was anticipated that this antibody might not be able to detect the truncated FNDC4 if it were present in human and/or rat brain lysates. However, we demonstrated that truncated FNDC4 could not be translocated to the cell membrane or glycosylated, resulting in rapid degradation, as for the unglycosylated canonical protein, which was barely detectable in brain samples (Figure 2H). As a result, the truncated FNDC4 protein is unlikely to be functional.

FNDC4 has been reported to be cleaved at the cell membrane with release of its extracellular portion as a soluble peptide (12). That conclusion was based on cDNA overexpression experiments like those we performed. Those authors overexpressed a FLAG-tag–fused (N-terminus) and MYC-tag–fused (C-terminus) mouse FNDC4 protein with an amino acid sequence that is very similar to that of human FNDC4 (Supplemental Figure 4). They also detected multiple bands of the overexpressed FNDC4 protein by Western blot analysis of cell lysates, just as we did for our human FNDC4 canonical protein (Figure 2B). In addition, they observed only a single band (~25 kD) in the cell culture media using antibody directed against an N-terminus–fused FLAG-tag (12). Those investigators concluded that the band detected in the media (~25 kD) represented a cleaved and secreted portion of their overexpressed FNDC4 protein because the band had a reduced MW compared with the cellular version (although without cell lysates as a control in the same blot) and was undetectable in the media when they tested antibody against a C-terminus–fused MYC-tag (12). However, those results did not exclude the possibility that the single band detected in cell culture media was an unglycosylated cytoplasm-enriched full-length FNDC4 that could be released into cell culture media, as we observed in similar experiments (Supplemental Figure 3E).

Taken as a whole, our results strongly suggest that the functional endogenous FNDC4 protein in the brain is primarily a TM glycoprotein. This observation led us to investigate FNDC4 function in the brain, as described subsequently.

### FNDC4 interacts with cell membrane proteins in neural progenitor cells.

**Figure 3:**
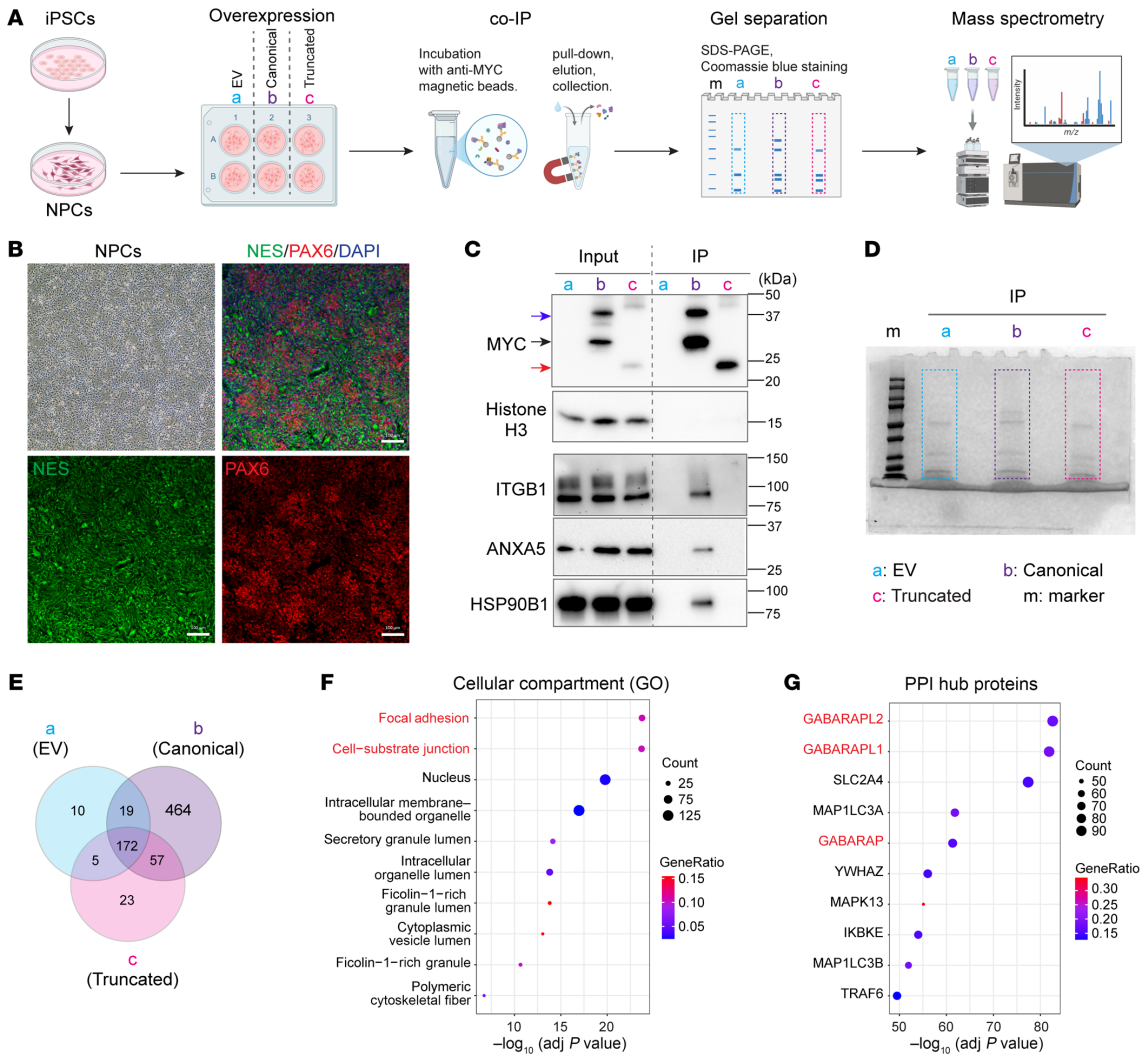
FNDC4-interacting proteins in NPCs. (**A**) Scheme for the identification of FNDC4-interacting protein in iPSC-derived NPCs by co-IP followed by MS. MYC-tagged FNDC4 proteins were overexpressed in NPCs. EV control (a), canonical (b), and truncated (c) FNDC4 proteins were overexpressed in the same batch of NPCs. Co-IP was performed using anti-MYC magnetic beads. Protein lysates for all 3 overexpression samples were separated by SDS-PAGE, and protein bands were visualized by Coomassie blue staining. Gel sections were cut for MS analysis. The figure was created with BioRender.com. (**B**) Characterization of NPCs by immunofluorescent staining of nestin (NES) and paired box 6 (PAX6), which are marker proteins for NPCs. Scale bars: 100 μm. (**C**) Western blots show successful pull-down of MYC-tagged canonical and truncated FNDC4 proteins that were overexpressed in NPCs. Histone H3 was blotted as an internal control for input samples. ITGB1, ANXA5, and HSP90B1 were only precipitated after pull-down of canonical FNDC4, indicating that they specifically interact with canonical but not truncated FNDC4 protein. (**D**) Separation of proteins in IP samples by SDS-PAGE and visualization of protein bands by Coomassie blue staining. Dashed boxes indicate gel sections that were cut for MS analysis. (**E**) Venn diagram overlapping MS-identified proteins in 3 IP samples. A total of 464 proteins were identified specifically in canonical FNDC4 IP sample. (**F** and **G**) The top 10 terms enriched in the GO Cellular Compartment (**F**) and in the PPI Hub Proteins (**G**) by 242 highly confident canonical FNDC4-interacting proteins.

The function of FNDC4 in the brain was unclear when we began our studies. However, it is a protein with a simple structure for which the major potential functional domain is an extracellular FN3 domain (Figure 1D). FN3 domains are known to bind to protein partners and to mediate cell–cell interaction (19). These facts led us to identify FNDC4-interacting proteins that might elucidate its function in the brain. Specifically, we performed FNDC4 co-IP, followed by mass spectrometry (MS) and pathway/term enrichment using the FNDC4-interacting proteins to help us study its function. Because of the unavailability of FNDC4 antibody for co-IP, we overexpressed Myc-FLAG–tagged FNDC4 proteins in iPSC-derived neural progenitor cells (NPCs) followed by pull-down of the overexpressed proteins using anti-MYC magnetic beads (Figure 3A).

Based on our experience with co-IP and MS analysis (20), we knew that nonspecific protein binding and protein contamination could happen during IP and MS experiments. Therefore, we included both empty vector (EV) and truncated FNDC4 overexpression as controls for the identification of canonical FNDC4-interacting proteins in our experiments. We used NPCs to identify FNDC4-interacting proteins because FNDC4 appeared to be functional before the neuron generation. Specifically, based on transcriptomic studies of stem cell–to–neuron differentiation, FNDC4 expression levels were significantly increased during the period from early NPC differentiation to the beginning of neuron generation and were sustained at similar levels in neurons (21), indicating that FNDC4 was required prior to the generation of neurons. These NPCs were characterized by immunostaining of markers (Figure 3B).

After a successful pull-down of overexpressed FNDC4 proteins (Figure 3C), proteins in the IP samples were separated by SDS-PAGE, and gel sections were cut for MS analysis (Figure 3D). The MS-identified peptides mapped to 206 proteins in EV, 712 proteins in canonical FNDC4, and 257 proteins in truncated FNDC4 samples. To filter out false positive results, we overlapped proteins identified in the 3 IP samples. Finally, 464 canonical FNDC4–specific interacting proteins were identified in NPCs (Figure 3E and Supplemental Table 2). These 464 proteins included CAV1, REEP3, and GHITM, all of which are known FNDC4-interacting proteins in other cell lines identified by the BioPlex Interactome (22) (Supplemental Figure 5).

We then performed pathway/term enrichment using those FNDC4-interacting proteins. To further enhance confidence in the input FNDC4-interacting proteins, the proteins with a total peptide count of 1 in the MS experiment (Supplemental Table 2) were not included as input since they might or might not have been identified by chance, depending on both their amino acid sequences and abundance. Finally, 242 high-confidence FNDC4-interacting proteins were used for pathway/term enrichment. Focal adhesion, cell-substrate junction, and a series of extra- and intracellular membrane–related terms were enriched as the top terms in the Cellular Component (Figure 3F) and Molecular Function aspects of the Gene Ontology (GO) (23) and COMPARTMENTS (24) analyses (Supplemental Figure 6). One protein that was consistently among the top cellular membrane–related terms was integrin subunit β-1 (ITGB1), a member of the integrin family that is known to bind fibronectin through the FN3 domain (the protein domain that gives FNDC4 its name) in extracellular matrix (25). ITGB1 is one of the most abundantly expressed β integrin subunits in human brain cortex (26), and it is known to play important roles in neurogenesis through interaction with other extracellular matrix proteins (27). We further validated the FNDC4 interactions with ITGB1 and with HSP90B1 and ANXA5 by Western blot (Figure 3C). Both HSP90B1 and ANXA5 are membrane-associated, extracellular vesicle marker proteins (28) that are involved in multiple neural functions, including neurodevelopment (29, 30). The pathways/terms enriched by FNDC4-interacting proteins matched FNDC4 membrane localization and suggested a role for FNDC4 in cell–cell communications through cell surface protein binding and/or extracellular vesicles.

Another interesting finding is the enrichment of Protein–Protein Interaction (PPI) hub proteins, which identifies PPI hub proteins for input proteins based on more than 50 known PPI partners (of the hub protein) extracted from literature-based databases (31). The top enriched PPI hubs (proteins) for FNDC4-interacting proteins include 2 GABA type A receptor-associated protein–like proteins (GABARAPL2 and GABARAPL1) and GABARAP itself (Figure 3G). GABARAP plays a crucial role in trafficking and synaptic clustering of GABA type A receptors (GABA_A_Rs) to the plasma membrane in neurons (32, 33). GABARAPLs, with a similar protein structure to GABARAP, could function similarly. For example, GABARAPL1 (also known as GEC1) increases cell surface expression of kappa opioid receptor (KOR) through facilitating its intracellular trafficking (34). This result suggested that FNDC4 could potentially interact with GABARAPs, which play roles in intracellular trafficking of the membrane receptors, including GABA_A_Rs and KOR. These receptors are involved in the mechanisms of action of AUD medicines (acamprosate and naltrexone) that have been used to treat patients recruited in our GWAS, which identified the *FNDC4* gene (9).

In summary, this series of experiments identified FNDC4-interacting proteins in NPCs, many of which are TM or membrane-associated proteins that play roles in neurodevelopment. Together with the fact that FNDC4 expression was significantly increased during the process of NPC-to-neuron differentiation (21), these results suggested a potential role for FNDC4 in neurogenesis.

### Generation and snRNA-seq analysis of FNDC4-KO neural organoids.

**Figure 4:**
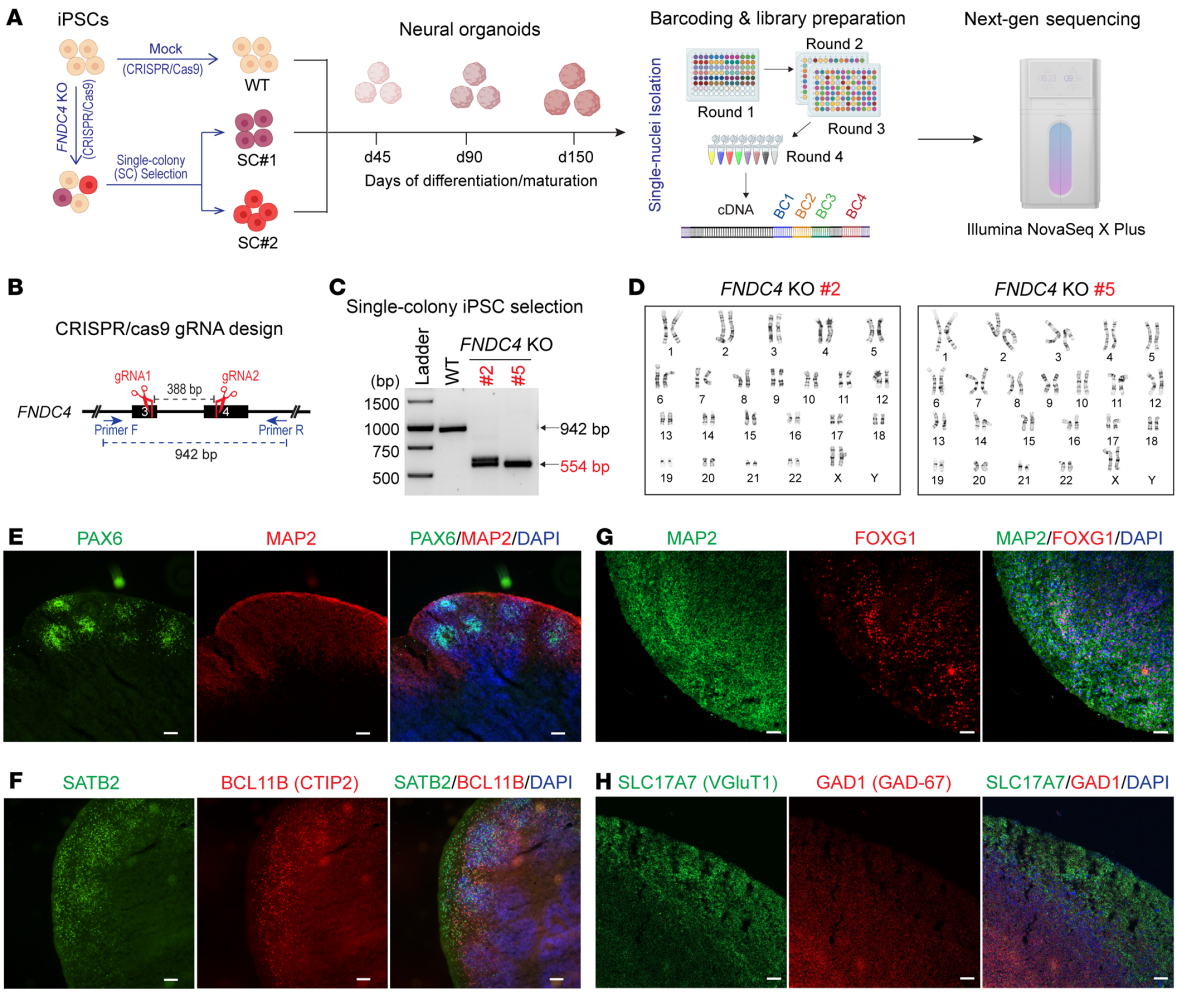
Generation of *FNDC4*-KO neural organoids for snRNA-seq analysis. (**A**) Depiction of experimental design. A human iPSC line was used to knock out the *FNDC4* gene by CRISPR/Cas9. Mock KO was performed without using gRNAs, which served as a WT control. Two homozygous KO single-colony (SC) iPSC lines were selected and differentiated to forebrain organoids as well as the WT iPSC. Three organoids generated from each iPSC line were harvested at 3 time points (days 45, 90, and 150). A total of 27 organoids (3 organoids × 3 iPSC lines × 3 time points) were used for single-nucleus isolation. RNA in these single nuclei was barcoded using split-pool combinatorial barcoding technology and were sequenced using the Illumina NovaSeq X plus platform. The figure was created with BioRender.com. (**B**) Design of gRNAs for *FNDC4* CRISPR/Cas9 editing and of PCR primers for *FNDC4*-KO colony selection. Successful CRISPR/Cas9 editing using both gRNAs was expected to remove 388 bp, resulting in a 554 bp amplicon by PCR using designed primers. (**C**) Agarose gel results showing the PCR amplicons using genomic DNA from the WT and 2 selected KO iPSC lines. (**D**) Both of the *FNDC4*-KO iPSC lines had their genomic integrity confirmed by karyotyping. See Supplemental Figure 7 for details of the iPSC single-colony selection, genotyping, and karyotyping. (**E**–**H**) Representative cryosections of forebrain organoids at day 150 of differentiation/maturation stained for markers of neural progenitor cells (PAX6) and neurons (MAP2) (**E**), superficial (SATB2) and deep (BCL11B, also known as CTIP2) neurons (**F**), developing forebrain neurons (MAP2 and FOXG1) (**G**), and glutamatergic (SLC17A7) and GABAergic (GAD1) neurons (**H**). Scale bars: 100 μm (**E** and **F**), 50 μm (**G** and **H**).

To further investigate the role of FNDC4 in neurogenesis, we studied FNDC4 function using human iPSC-derived neural organoids, a well-established CNS model for the study of human neurodevelopment (35, 36). Specifically, we generated *FNDC4*-KO iPSCs by CRISPR/Cas9 and differentiated both WT and homozygous *FNDC4*-KO iPSCs to generate dorsal forebrain organoids. The organoids were collected and studied at different time points (45, 90, and 150 days) of differentiation/maturation, and they were also analyzed by snRNA-seq (Figure 4A).

To create *FNDC4*-KO iPSCs, we designed 2 gRNAs to remove 388 bp and cause a frameshift in the ORF (Figure 4B). After CRISPR/Cas9 editing, selection of single-colony *FNDC4*-KO iPSC lines was performed (Supplemental Figure 7). Two of the *FNDC4*-KO iPSC lines that maintained a homozygous KO genotype after 3 passages were selected for further study (#2 and #5 in Figure 4C) and characterized by karyotyping to ensure genomic integrity (Figure 4D).

Both *FNDC4*-KO iPSC lines and WT iPSCs were then differentiated to generate dorsal forebrain organoids based on an established protocol (37) (Supplemental Figure 8A). We used dorsal forebrain organoids because FNDC4 is most highly expressed in human brain cerebral cortex (10) and because the prefrontal cortex is one of the brain reward regions, which play important roles in the neurobiology of addiction (38). The protocol that we used can reliably and reproducibly generate dorsal forebrain organoids with a rich diversity of cell types appropriate for human cerebral cortical tissue (37). The WT and *FNDC4*-KO iPSC-derived dorsal forebrain organoids were visually similar in their morphological shape (Supplemental Figure 8B). We further investigated their structure based on immunostaining of neural markers (Figure 4, E–H), all of which were comparable to those used to illustrate the original publications (37) with regard to these organoids.

To explore their molecular characteristics, the organoids were further analyzed by snRNA-seq. Specifically, 3 organoids for each experimental condition were pooled for 1 single-nuclei sample. Nine single-nuclei samples that matched with 9 experimental conditions (3 iPSC lines × 3 time points) were prepared. Nuclei from the 9 samples were barcoded using split-pool combinatorial barcoding technology, which allows sample multiplexing (39). Because all 9 samples were barcoded and sequenced in a single experiment, comparisons across samples could be controlled with minimal batch effects.

**Figure 5:**
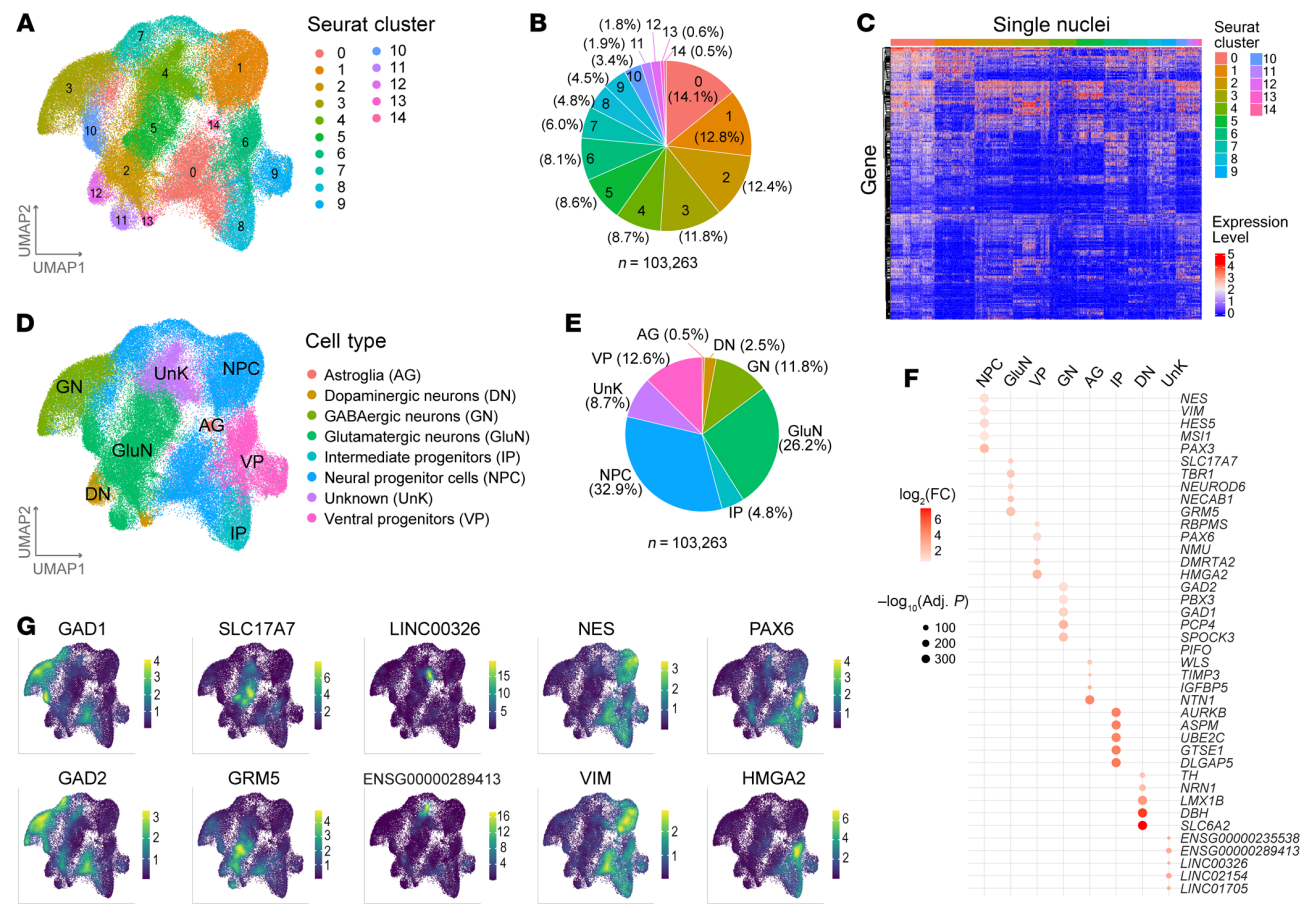
snRNA-seq analysis of iPSC-derived neural organoids. (**A**) UMAP visualization of snRNA-seq data for 27 forebrain organoids under 9 experimental conditions. Each dot represents a single nucleus (*n* = 103,263). Nuclei were clustered based on the similarity of their transcriptomic profiles (Seurat clustering). (**B**) Pie chart showing proportions of single nuclei in each Seurat cluster. (**C**) Heatmap showing the expression levels of genes that were differentially expressed across different Seurat clusters (color-coded and labeled in the top panel). Each row is a gene, and each column is a single nucleus. For visualization, 5,000 single nuclei were randomly pulled from the single-nuclei pool (*n* = 103,263) but with the maintenance of proportions of individual Seurat clusters, and the top 100 DEGs in each Seurat cluster based on adjusted *P* values (Bonferroni correction) were used to plot the heatmap. (**D**) Clustered single cells (*n* = 103,263) were annotated to neural cell types based on the differential expression of marker genes. A total of 8 cell types were annotated and color-coded. (**E**) Pie chart showing proportions of single cells for each annotated cell type. (**F**) Bubble plot showing top 5 differentially expressed marker genes for each annotated cell type. Each line is a gene marker, and each column is an annotated cell type. (**G**) UMAP plots illustrating the expression levels of the classic marker genes for cell type annotation. The colors from dark to bright (yellow) represent the expression level from low to high.

A total of 154,955 single nuclei from 9 samples were sequenced with a mean sequencing depth of approximately 20K reads/cell. Single nuclei with fewer than 500 detected genes were filtered out, leaving 103,263 single nuclei for further analysis. The snRNA-seq identified 15 cell clusters (Figure 5A), with proportions ranging from 0.5% to 14.1% of total single nuclei (Figure 5B), based on the single-nuclei transcriptomic profiles (Figure 5C). These 15 cell clusters were then annotated to 8 cell types based on the expression of marker genes in each cluster (Figure 5D). The majority of the cells in those dorsal forebrain organoids were annotated as progenitor cells and neurons, specifically GABAergic (GN) or glutamatergic (GluN) neurons (Figure 5E), both of which are the major neuron types present in human brain cerebral cortex. The top differentially expressed marker genes included classical markers for GNs (*GAD1* and *GAD2*), GluNs (*SLC17A7*), and NPCs (*NES* and *VIM*) (Figure 5, F and G).

### FNDC4 KO alters glutamatergic and GABAergic neurogenesis and their relative balance in neural organoids.

**Figure 6:**
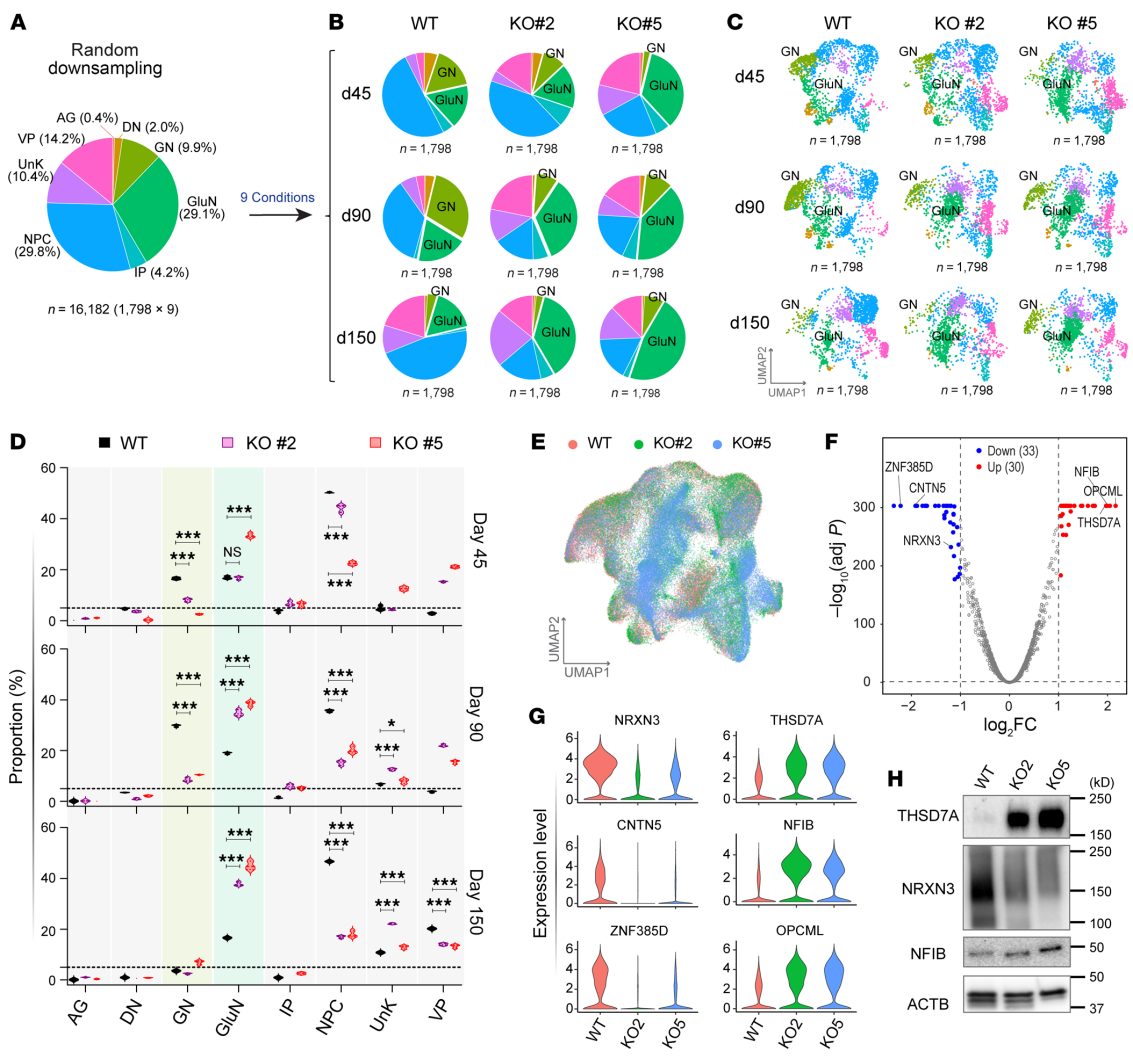
*FNDC4* KO led to an increased population of glutamatergic neurons defined by snRNA-seq. (**A**) Pie chart showing proportions of single cells for each annotated cell type in random downsampling single-nuclei samples under 9 experimental conditions (*n* = 16,182). Random downsampling was performed to ensure that each of the 9 experimental conditions included the same number of single nuclei (*n* = 1,798) for comparison of cell type proportions. (**B**) Pie charts showing the proportions of annotated cell types in dorsal forebrain organoids for 9 individual experimental conditions (WT, KO#2, and KO#5 at days 45, 90, and 150 of organoid differentiation/maturation). (**C**) The single nuclei from 9 individual experimental conditions were also visualized by UMAP plots. (**D**) Violin plots comparing proportions of 8 cell types in WT and 2 *FNDC4*-KO organoids across 3 time points after 3 rounds of random downsampling. *P < 0.05, ***P < 0.001, 2-way ANOVA with Dunnett’s multiple comparisons to WT samples. *P* values for cell types with a proportion of 5% (dashed line) or less are not shown. (**E**) UMAP showing distributions of single-cell (*n* = 103,263) clusters from WT and 2 *FNDC4*-KO organoids. (**F**) Volcano plots for DEGs in pseudo-bulk RNA-seq analysis comparing *FNDC4* KO to WT organoids. DEGs were defined by |log_2_FC| ≥ 1 and adjusted *P* < 0.05. (**G**) Violin plots showing the expression of 6 top DEG functions in neurogenesis in WT and 2 *FNDC4*-KO organoids. (**H**) Western blot validation of protein levels for 3 DEGs using protein lysates obtained from WT and *FNDC4*-KO organoids on day 150. ACTB was blotted as a loading control.

Our snRNA-seq data allowed us to compare the results across 9 experimental conditions. To ensure that the same number of single nuclei were compared for each condition, random downsampling was performed to match the single nuclei numbers in all 9 conditions. The proportions of each cell type after random downsampling (Figure 6A) were similar to that in the total cell pool (Figure 5E). However, when compared with WT organoids, there was a significant increase in the number (or proportion) of GluNs and a significant decrease in the number of GNs in the *FNDC4*-KO organoids (Figure 6, B and C). This observation was consistent in 2 single-colony *FNDC4*-KO iPSC-derived organoids at multiple time points. We repeated the random downsampling 3 times, and each time observed the same result, suggesting that these results had not occurred by chance (Figure 6D and Supplemental Table 3).

To avoid the limitation of available single nuclei number (*n* = 1,798) for the WT day 150 condition, we removed the day 150 time point, thus making it possible to compare 7,305 single nuclei per condition across 6 conditions (Supplemental Figure 9 and Supplemental Table 4). We once again observed a significant increase in the number of GluNs and, simultaneously, a decrease in the number of GNs in the *FNDC4*-KO iPSC-derived organoids when compared with the WT organoids.

We further compared the transcriptomic profiles of KO versus WT organoids by pseudo-bulk RNA-seq analysis of the single-cell transcriptomes (Figure 6E). A total of 63 differentially expressed genes (DEGs) were identified (Figure 6F and Supplemental Table 5). Top DEGs included *NIFB*, *THSD7A*, *NRXN3*, *CNTN5*, and *OPCML* (Figure 6G), genes with known roles in neurodevelopment and/or neurogenesis (40–44). Some of the DEGs, including *THSD7A*, *NRXN3*, *OPCML*, and *ZNF385D*, have been associated with alcohol consumption or sensitivity to alcohol by GWAS (45–47). We also validated some of the top DEGs regarding their protein levels, all of which showed a consistent result with the DEG analysis (Figure 6H).

Taking together, these neural organoid studies demonstrated that *FNDC4* KO altered the glutamatergic and GABAergic neuron proportions in opposite directions, a result that further supported the role of FNDC4 in neurogenesis.

### Transcriptomic profile of FNDC4-dependent neurogenesis.

**Figure 7:**
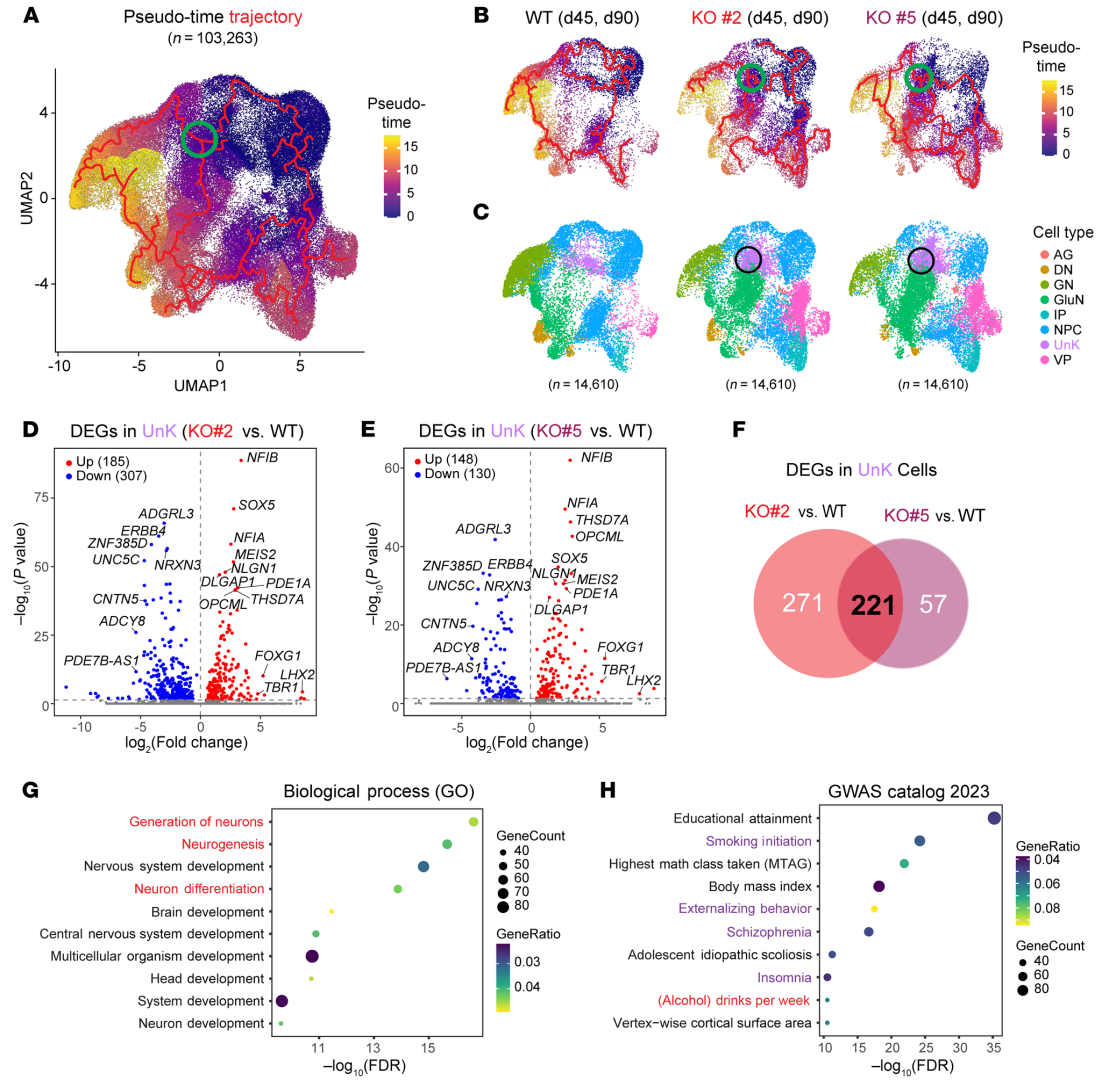
Transcriptome profile of FNDC4-dependent neurogenesis. (**A**) UMAP visualization inferring the order of cells along neurodevelopmental trajectories. Transcriptomic data for all single nuclei (*n* = 103,263) were included in the analysis. Each cell/(dot) was assigned a pseudo-time value (color-coded) that represents which stage the cell is in along neurodevelopmental trajectories. Cells at later stages of neurodevelopment (higher pseudo-time value) are colored yellow. Trajectories for neurodevelopment are depicted as red lines. Branch spots leading to different neuronal subtypes are circled in green. (**B**) Single-cell trajectory analysis using single-nuclei transcriptomics of forebrain organoids differentiated from the WT, *FNDC4*-KO#2, and *FNDC4*-KO#5 iPSC lines. Random downsampling was performed to ensure that the same number of cells (*n* = 14,610) from WT and KO organoids was included for comparison. Single nuclei of organoids differentiated from the same iPSC lines at 2 time points (days 45 and 90) were combined to increase diversity (in developmental stages) and number of single cells for trajectory analysis. The branch point in the trajectories leading to different neuronal subtypes (circled in green) was observed in KO but not in WT organoids. (**C**) UMAP visualization of the same single nuclei as in **B** with cell types color-coded. The branch point observed in KO was overlapped with a cluster of UnK cells. (**D** and **E**) Volcano plots for DEGs in UnK cells when comparing KO to WT organoids. (**F**) Venn diagram showing 221 common DEGs in UnK cells that were identified in organoids differentiated from both KO iPSC lines. (**G** and **H**) Top terms enriched by 221 DEGs in UnK cells after *FNDC4* KO in the GO Biological Process (**G**) and GWAS catalog (**H**) phenotype/trait enrichment analyses. The FDR for pathway enrichment was computed from Fisher’s exact test and adjusted using the Benjamini-Hochberg method for the correction for multiple hypotheses testing.

We next explored the potential molecular mechanism(s) involved in FNDC4-dependent neurogenesis. Based on all available single-nuclei transcriptomes, pseudo-time trajectories for neural development from less mature NPCs to more mature neurons were generated (Figure 7A). The pseudo-time trajectory analysis identified a branch point that led to distinct paths that generated GNs or GluNs (Figure 7A, green circle). When comparing the trajectories of WT and *FNDC4* KO organoids, this branch point could only be clearly identified in *FNDC4*-KO but not in WT organoids (Figure 7B, green circles). Furthermore, this branch point overlapped a cell population of unknown (UnK) cells (Figure 7C) for which the transcriptome profiles did not match any known neural cell types.

We next compared the transcriptomes of UnK cells in *FNDC4*-KO organoids with those in WT organoids to identify genes involved in the cellular decision-making process for glutamatergic and GABAergic neurogenesis. DEGs with fold changes (FCs) more than 2 (|log_2_FC| ≥ 1, adjusted *P* < 0.05) in the UnK cells of each *FNDC4*-KO organoid compared with WT were identified (Figure 7, D and E). A total of 221 DEGs were consistently identified in *FNDC4*-KO organoids derived from both single-colony iPSC lines (Figure 7F and Supplemental Table 6).

Many of the top DEGs in UnK cells, including *NIFB*, *THSD7A*, *OPCML*, *NRXN3*, *CNTN5*, and *ZNF385D* (Figure 7, D and E), were also top DEGs in pseudo-bulk RNA-seq analysis for organoids (Figure 6F). We also noticed that many genes that were not DEGs in pseudo-bulk RNA-seq analysis for organoids became significant DEGs in UnK cells. For example, 3 genes, *FOXG1*, *TBR1*, and *LHX2*, were dramatically increased in UnK cells after *FNDC4* KO (log_2_FC > 5; Figure 7, D and E). All 3 of these DEGs encode transcription factors that play important roles in regulating GABAergic and/or glutamatergic neurogenesis (48–50). Both *FOXG1* and *TBR1* also maintain high expression levels in mature cortical neurons (51). *FNDC4* KO resulted in a dramatic change in expression of these 3 genes in UnK cells, a cell type that represents a transition status when cells are committed to become either GN or GluN cells.

The 221 *FNDC4*-dependent DEGs in UnK cells were used to perform GO enrichment to explore the biological function of FNDC4 in neural organoids. The top enriched terms by GO Biological Process analysis were all related to neurogenesis (Figure 7G), a result consistent with our observation of dysregulated GABAergic and glutamatergic neurogenesis in forebrain organoids after *FNDC4* KO (Figure 6). The GO Cellular Component and Molecular Function analyses have enriched terms such as cell junction, synaptic membrane, neuron-to-neuron synapse, and cell adhesion molecule binding (Supplemental Figure 10), results that were consistent with the pathway enrichment using FNDC4-interacting proteins identified in NPCs, suggesting a molecular function for FNDC4 that is involved in neural cell–cell interaction. Finally, the 221 DEGs were also used for GWAS catalog (52) enrichment to help enhance our understanding of the potential role of the brain expression of FNDC4 in clinical phenotypes/traits. Alcohol consumption (drinks per week) and multiple behavioral/neuropsychiatric phenotypes that often coexist with AUD (smoking initiation, externalizing behavior, schizophrenia, and insomnia) were significantly enriched (Figure 7H), supporting a possible functional role for brain expression of FNDC4 in AUD.

In summary, using snRNA-seq data generated using WT and *FNDC4* KO forebrain organoids, this series of analysis suggested that *FNDC4* is involved in AUD-related phenotypes by regulating glutamatergic and GABAergic neurogenesis, perhaps through its function in binding to cellular membrane proteins and mediating cell–cell interactions.

### FNDC4-KO neural organoids exhibit higher neuronal excitability.

**Figure 8:**
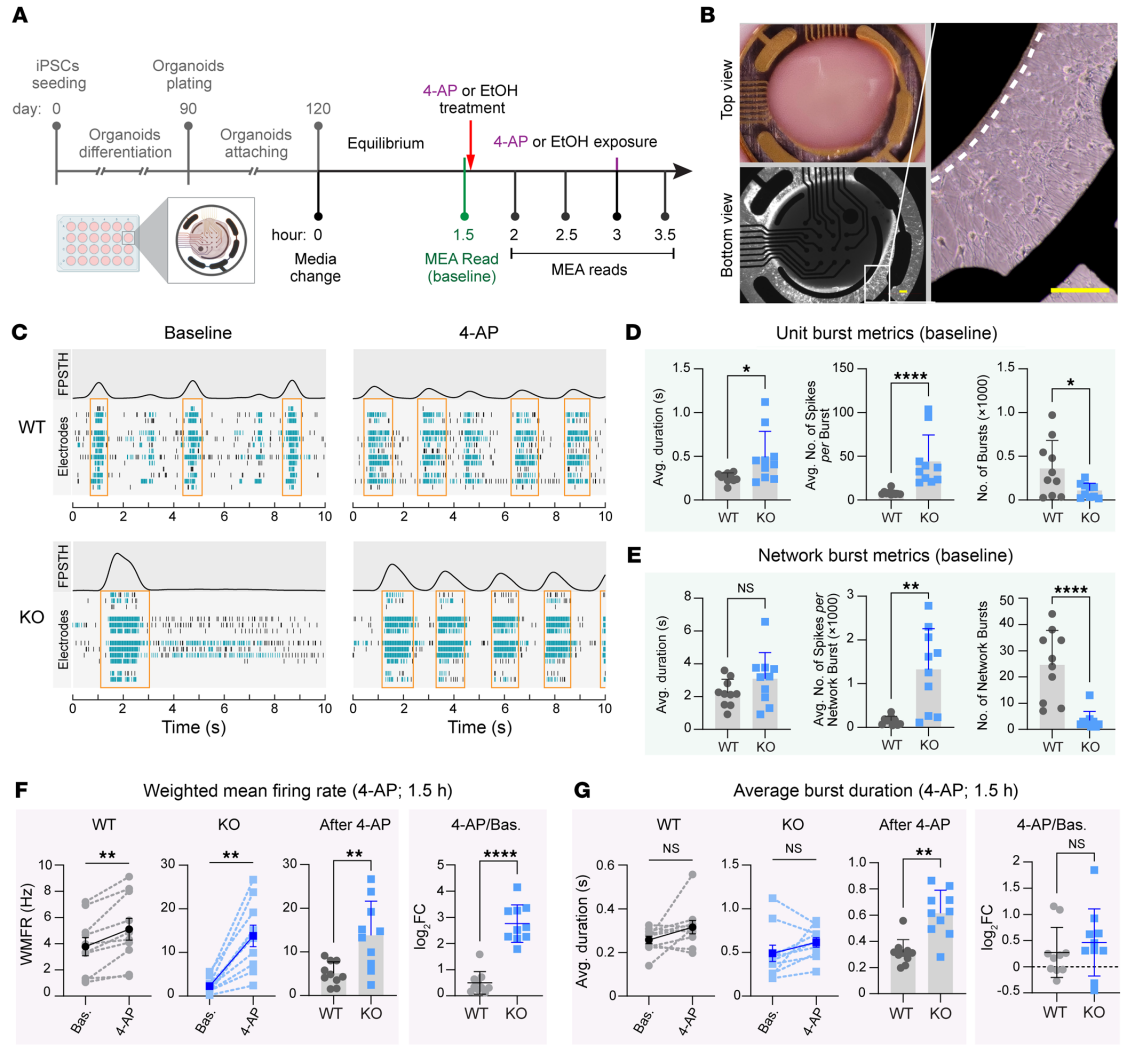
Electrical activities of WT and *FNDC4*-KO neural organoids. (**A**) Scheme for the MEA assay of iPSC-derived dorsal forebrain organoids. See Supplemental Methods for detailed description. (**B**) Pictures showing attachment of a neural organoid to the electrodes in a well of the MEA plate. Zoomed-in picture shows the edges (dashed lines) of attached organoids, and neural cells are visible around the edge. Scale bars: 200 μm. (**C**) Raster plots showing the detected neuronal spikes by electrodes in a well of WT or KO organoids before and after 4-AP treatment. Each tick indicates a spike detected by an electrode, and each row indicates the electrode. Green ticks indicate that the spikes were part of a burst, while black ticks are not. Above the raster is a FPSTH, illustrating the total number of spikes detected by all electrodes at each time. Plots show the first 10 of 180 seconds of recording. (**D** and **E**) Scatterplots showing the baseline unit burst (**D**) and network burst metrics (**E**) of the WT and *FNDC4*-KO organoids (*n* = 10/group). Each dot/square represents a single organoid. (**F** and **G**) The WMFR (**F**) and the average burst duration (**G**) of organoids measured before (Bas.) and after 4-AP exposure were compared. Values for individual organoids before and after 4-AP treatment are paired by dashed lines. Solid lines link values of mean ± SEM. **P < 0.01, Wilcoxon’s matched-pair, signed-rank test. Values after 4-AP exposure and the log_2_FC values after 4-AP exposure (4-AP/Bas.) are also shown in scatterplots, with each dot/square representing a single organoid. In **D**–**G** (except for plots with values paired by dashed lines in **F** and **G**), data are shown as the mean ± SD. *P < 0.05, **P < 0.01, ****P < 0.0001, Mann-Whitney test.

We next measured electrical activities of the WT and *FNDC4* KO forebrain organoids using the MEA assay. Specifically, we repeated the generation of WT and *FNDC4*-KO forebrain organoids from iPSCs for MEA assay (see Figure 8A for the scheme). After organoids were cultured in the MEA plate for 30 days, neural cells emerged from the edges of organoids to plate, indicating established attachment to the plate (Figure 8B).

The MEA plate with organoids was recorded continuously for up to 3 minutes. Representative MEA readouts (first 10 seconds) for a WT and a KO organoid, before and after the 4-aminopyridine (4-AP) exposure, are shown in Figure 8C. The 4-AP is a nonselective potassium channel blocker that has been widely used to induce neuronal excitability in in vitro models including neural organoids (53). A neuronal spike detected by each electrode at a time is indicated by a tick. Green ticks indicate that the spikes are part of a unit burst, while black ticks are not. Unit bursts that occurred at the same time among multiple electrodes were counted as network bursts (Figure 8C, orange rectangles), which reflect the functional neural networks of those organoids. The total number of spikes occurring throughout all electrodes at each time was summed and visualized as a filtered population-spike-time histogram (FPSTH) above the ticks. A network burst often gives a peak in the FPSTH (Figure 8C).

When comparing WT and *FNDC4*-KO organoids (*n* = 10/group) at baseline, there was no significant difference in their electrical activities, including the total number of spikes and the weighted mean firing rates (WMFRs) during the 3-minute recording (Supplemental Figure 11A). However, the KO organoids had significantly longer average burst duration than the WT (Figure 8D). Longer bursts indicate more excitation or less inhibition, as it takes longer to shut down a burst. Consistently, the KO organoids also have significantly more spikes per burst and less total number of bursts during the 3-minute recording (Figure 8D). We further compared the network burst metrics that showed a similar result to the unit burst metrics (Figure 8E). Although not statistically significant, the KO organoids tended to have a longer average network burst duration than the WT (Figure 8E). In addition, the network bursts in KO organoids were stronger and better synchronized than the WT, as indicated by significant higher burst peak and synchrony index, respectively (Supplemental Figure 11B). The MEA data demonstrated that *FNDC4*-KO forebrain organoids exhibit higher neuronal excitability than the WT, a result that is consistent with the snRNA-seq data showing increased glutamatergic and decreased GABAergic neuron proportions in *FNDC4*-KO organoids.

We next attempted to study how the WT and *FNDC4*-KO organoids respond to ethanol (EtOH) exposure using MEA assay. To establish the treatment experiments, we first exposed the organoids to 4-AP. As expected, 1.5 hours of 4-AP exposure induced electrical activities in the neural organoids, with significantly increased WMFRs in both WT and KO organoids, and the KO organoids showed a more significant increase in WMFRs with a higher log_2_FC after 4-AP stimulus (Figure 8F). However, 4-AP exposure did not significantly change the average burst duration in the organoids. The KO organoids maintained a longer burst time after 4-AP exposure (Figure 8G). This result is consistent with the 4-AP mechanism of action, which does not directly affect the balance of excitatory and inhibitory neurotransmission (54) and thus is not expected to significantly change the burst duration. Using 4-AP as a control, we not only established drug treatment experiments for MEA assay but also enhanced the observation made at baseline that *FNDC4*-KO organoids exhibit higher neuronal excitability than the WT.

To our knowledge, there is no previous report using MEA assay to measure electrical activity of neural organoids in real-time EtOH exposure. Therefore, we first optimized experimental conditions by testing multiple EtOH concentrations and exposure times using individual WT organoids. The result suggested that the organoids could respond to EtOH at 12.5 mM (approximately a blood alcohol concentration of 0.06%) within the first 2 hours of exposure in terms of their electrical activity (Supplemental Figure 12A). We then exposed more WT and KO organoids to 12.5 mM EtOH and recorded their electrical activities every 30 minutes until 2 hours. The KO organoids showed decreased electrical activity in response to EtOH exposure, as their mean log_2_FC in WMFR was below 0 (Supplemental Figure 12B). However, there was no significant difference between the WT and KO groups. Like the 4-AP exposure, no significant changes in average burst duration were observed in either WT or KO organoids after EtOH exposure, suggesting that acute EtOH exposure (within 2 hours) did not change the balance of excitatory and inhibitory neurotransmission.

In summary, we performed MEA assays to measure the electrical activity of WT and *FNDC4* KO forebrain organoids. The *FNDC4*-KO organoids exhibited significantly longer neuronal burst durations than the WT, indicating more excitation or less inhibition in those KO organoids. This result supports the conclusion that FNDC4 can affect the functional neuronal networks, at least in part, through its role in neurogenesis.

## Discussion

In the present study, we characterized a genetic locus associated with AUD-related phenotypes, suggesting an FNDC4-dependent neurogenesis mechanism that contributes to AUD pathophysiology. Our observation that *FNDC4* KO resulted in dysregulated GABAergic and glutamatergic neurogenesis is consistent with the current understanding of AUD pathophysiology and the mechanism of action of drugs used to treat AUD. Alcohol directly interacts with GABA and glutamate receptors, and it is believed that AUD develops as a result of disrupted homeostasis of neurotransmission after exposure to alcohol (3). AUD pharmacotherapy is also targeted to neurotransmission mechanisms.

Our results suggest a possible scenario in which individual adults might have differing ratios of GABAergic and glutamatergic neurons, and those interindividual differences in neuron populations might be, at least in part, due to genetic polymorphisms related to the *FNDC4* gene that are linked to individual variation in AUD risk and drug treatment response. Specifically, our GWAS showed that variant SNP alleles that were associated with increased FNDC4 splicing isoforms, which encode a loss-of-function truncated FNDC4 protein (Figure 2), were also associated with adverse drug treatment response (Figure 1). Since FNDC4 loss of function resulted in increased glutamatergic but decreased GABAergic neurogenesis (Figure 6), our results raise the possibility that patients with AUD who carry the variant SNP allele (with increased expression of the FNDC4 splicing isoform) might benefit from therapy that antagonizes glutamatergic neurotransmission. As a result, our studies may provide a genetic basis for individualized AUD treatment.

AUD often coexists with other substance use disorders, particularly smoking, and with mental health disorders (2). The FNDC4 function in glutamatergic and GABAergic neurogenesis supports the possibility that *FNDC4* might be a pleiotropic gene that contributes to additional neuropsychiatric disorders. Specifically, the FNDC4-dependent DEGs identified in our neurodevelopment trajectory analysis have enriched smoking initiation and schizophrenia as clinical phenotypes, and several top FNDC4-dependent DEGs are known risk genes for different psychiatric disorders (Figure 7). For example, the *THSD7A* gene, for which expression is significantly increased after *FNDC4* KO (Figure 6 and 7), has been identified in GWAS for bipolar disorder (BD) risk (55) and anticonvulsant treatment response in BD (56). In addition, a genetic locus (index SNP, rs12474906) that is an expression QTL for *FNDC4* (10) has been identified by large-cohort GWAS as a pleiotropic locus for 8 different psychiatric disorders (*P* = 8 × 10^–9^) including schizophrenia, BD, and attention-deficit/hyperactivity disorder (57), all of which often co-occur with AUD (58–60).

The fact that *FNDC4* KO leads to increased glutamatergic but decreased GABAergic neurogenesis is also compatible with observations made in *Fndc4*-KO mice that display hyperactivity (61). Hyperactive behavior was found to be related to an imbalance between inhibitory and excitatory neuron development (62) as well as increased glutamatergic transmission (63). Notably, childhood attention-deficit/hyperactivity disorder is a substantial risk factor for the development of alcohol and substance use disorders (64).

Finally, there are limitations of our studies, beginning with our use of in vitro CNS models. We used human iPSC-derived dorsal forebrain organoids to study FNDC4 molecular function because FNDC4 is expressed more highly in human brain cerebral cortex than in other brain regions (GTEx) (10). To our knowledge, FNDC4 function in human CNS models has not been reported previously. However, FNDC4 is also expressed in other brain regions. Its function in other brain regions needs to be studied further. Although iPSC-derived neural organoids provide an opportunity to manipulate human gene expression in brain-like models and to capture neural cell–cell interactions during neural development, this model obviously does not fully capture neural function in the adult human brain. Therefore, FNDC4 neural functions other than neurogenesis would not necessarily be captured in our study using neural organoids. Although behavioral changes in hyperactivity, a risk factor for developing AUD, have already been observed in *Fndc4*-KO mice, whether those mice develop alcohol-related behaviors is currently unknown and needs to be studied. The animal models might also provide an opportunity to test novel therapeutic interventions targeting FNDC4-related mechanisms in future studies.

In summary, we have studied the function of the *FNDC4* gene, for which sQTLs in the brain have been associated with AUD risk and variation in drug treatment response (9). We demonstrated that the *FNDC4* sQTL-related mRNA splice isoform results in loss of function of *FNDC4*, which can then lead to the dysregulation of the balance between glutamatergic and GABAergic neurogenesis. These results strongly support the possibility that FNDC4 function in the CNS might play a role in AUD risk and variation in response to the drug treatment of AUD, providing a genetic basis for individualized AUD pharmacotherapy and an opportunity for the development of novel AUD therapies.

## Methods

### Sex as a biological variable.

Sex was not considered a biological variable in this study.

Detailed methods, including experimental design, protocols, and data analysis, are presented in Supplemental Methods. Information for all reagents and key resources, including antibodies, cell lines and culture media, plasmids, and datasets, is listed in Supplemental Table 1.

### Statistics.

Statistical analysis is specified in the figure legends. A *P* value less than 0.05 was considered significant.

### Study approval.

All participants in the GWAS provided informed consent for the use of their clinical data and DNA in genetic research related to AUD and treatment outcomes, and the study received approval from the Mayo Clinic Institutional Review Board. Animal tissues were harvested in accordance with the NIH Guidelines for the Care and Use of Laboratory Animals and approved by the Mayo Clinic Institutional Animal Care and Use Committees.

### Data availability.

The snRNA-seq data generated in this study have been deposited in NCBI’s Gene Expression Omnibus and are accessible through accession number GSE285126. Values for all data points in graphs are reported in the Supporting Data Values file.

## Author contributions

XZ and DL designed the study. XZ, SK, Li Wang, ED, JZ, AS, AJ, CMW, IM, MW, CC, AA, TMK, and DL performed the experiments and collected the data. XZ, AJJ, IMG, HG, and DL analyzed the data. DL, XZ, AJJ, SK, and RMW wrote the manuscript. BJC, NS, Liewei Wang, MAF, JMB, VMK, and HL interpreted the results and revised the manuscript. DL and RMW supervised the project. XZ, AJJ, and SK contributed equally to this work, and their authorship order reflects their timing of participation in the study.

## Funding support

This work is the result of NIH funding, in whole or in part, and is subject to the NIH Public Access Policy. Through acceptance of this federal funding, the NIH has been given a right to make the work publicly available in PubMed Central.

- NIH grants R01AA027486, R01GM028157, R21AA030184, and U01DA055017.
- Mayo Foundation for Medical Education and Research.
- SK and AA were supported by NIH T32 Training Grant in Clinical Pharmacology (T32GM08685).

## Supplementary Material

Supplemental data

Unedited blot and gel images

Supplemental tables 1-6

Supporting data values

## Version 1 01/08/2026

In-Press Preview

## Version 2 03/02/2026

Electronic publication
