# Supplementary material for: Alcohol use disorder–associated gene FNDC4 alters glutamatergic and GABAergic neurogenesis in neural organoids
Source: J Clin Invest. 2026 Jan 8;136(5):e193204. doi: 10.1172/JCI193204 (PMC12948423; doi:10.1172/JCI193204)

Supplemental Materials

Alcohol Use Disorder-Associated Gene *FNDC4* Alters Glutamatergic and GABAergic Neurogenesis in Neural Organoids

Authors:
Xiujuan Zhu[1*], August J. John[1*], Sooan Kim[1*], Li Wang[1], Enci Ding[1], Jing Zheng[1], Ateka Saleh[1], Irene Marín-Goñi[1,5], Abedalrahman Jomaa[1], Huanyao Gao[1] , Meijie Wang[1], Ching Man Wai[2], Irene Moon[1], Cindy Chen[1], Alireza Agahi[1], Brandon J. Coombes[3], Tony M. Kerr[1], Nobuyoshi Suto[1], Liewei Wang[1], Mark A. Frye[4], Joanna M. Biernacka[3,4], Victor M. Karpyak[4], Hu Li[1], Richard M. Weinshilboum[1,#], and Duan Liu[1,#]

Affiliations:
[1]Department of Molecular Pharmacology and Experimental Therapeutics, Mayo Clinic, Rochester, MN. U.S.A.
[2]Department of Biochemistry and Molecular Genetics, Feinberg School of Medicine, Northwestern University, Chicago, IL, USA
[3]Department of Quantitative Health Sciences, Mayo Clinic, Rochester, MN, U.S.A.
[4]Department of Psychiatry and Psychology, Mayo Clinic, Rochester, MN, U.S.A.
[5]Current Affiliation: Computational Biology and Translational Genomics, CIMA University of Navarra, Pamplona, Spain
[*]These authors contributed equally to this work.
[#]Corresponding authors

Supplemental Methods: Pages 1-9

References: Page 10

Supplemental Figures: Pages 11-22

Supplemental Methods

Cell Culture. HEK293T cell lines were obtained from the ATCC. HEK293T cell lines were cultured in DMEM with 10% FBS (see Supplemental Table S1 for detailed information of all reagents and key resources). A human episomal iPSC line was obtained from ThermoFisher. After directed differentiation and teratoma analyses, the iPSCs retained their differentiation potential for the ectodermal, endodermal, and mesodermal lineages. All cells tested negative for virus including HIV, HTLV, HSV, CMV, EBV, HBV and HCV. Cytogenetic analysis demonstrated an apparently normal female karyotype. Human iPSCs were cultured in StemFlex™ medium following the manufacture's protocol. NPCs were differentiated from iPSCs using STEMdiff™ SMADi Neural Induction Kit with the monolayer culture protocol (STEMCELL) and were then cultured in the Neural Progenitor Medium until use.

FNDC4 Overexpression. The human *FNDC4* cDNA plasmid construct was purchased from OriGene. This construct overexpresses a canonical FNDC4 protein fused with a MYC-FLAG-tag at the C-terminus. The *FNDC4* splice variant cDNA was synthesized by GenScript and was cloned into the same vector (pCMV6-Entry) as the *FNDC4* cDNA plasmid purchased from OriGene. HEK-293T cells were transfected with *FNDC4* cDNA constructs by using Lipofectamine™ 3000 Transfection Reagent. Eight hours after transfection, the cell culture media was replaced with fresh media. After ~48 hours of transfection, cells were harvested for protein extraction using Pierce™ IP Lysis buffer. Protein concentrations were determined by using the Pierce™ BCA Protein Assay Kit.

Animals. Male Wistar rats (350-375g) were purchased from Charles River Laboratories (Wilmington, MA). The tissues from the cortical and subcortical brain regions included caudate

putamen (CPU), nucleus accumbens (NAC), amygdala (AMG), lateral orbitofrontal cortex (lOFC), infralimbic/prelimbic cortices (IL/PL), anterior cingulate cortex (ACC), and "extra'" forebrain regions including medial orbitofrontal and motor cortices (Extra) that were harvested from three rats. Immediately after harvesting, all tissues were kept on dry ice and then stored in a -80°C freezer until further processing. For protein lysates preparation, those rat brain tissues were exposed to IP lysis buffer at a w/v ratio of 1mg/10μL followed by homogenization using a ½ mL syringe. Protein concentrations were determined by BCA assay. Animal tissues were harvested in accordance with the U.S. National Institutes of Health Guidelines for the Care and Use of Laboratory Animals and approved by the Mayo Clinic Institutional Animal Care and Use Committees (IACUC), Rochester, MN.

Western Blot. Denatured proteins were loaded onto a 4–20% Mini-PROTEAN® TGX™ Precast Protein Gels (Bio-Rad) to separate proteins. Precision Plus Protein Dual Color Standards (Bio-Rad) were used as protein markers for all blots included in this study. Proteins were transferred from the gels to PVDF membranes which were then blocked with 5% non-fat milk at room temperature for 1 hour. After washing with TBST, membranes were incubated with primary antibody (see Supplemental Table S1 for detailed information), which was dissolved in 1% BSA prepared in TBST at 4°C overnight with gentle rocking. Following incubation, the membranes were washed vigorously three times in TBST buffer and were then incubated with horseradish peroxidase (HRP)-labelled secondary antibody, which was dissolved in 5% non-fat milk at room temperature for 1 hour. The SuperSignal West Dura Extended Duration Substrate (ThermoFisher) was applied to the membranes, and radiographic images were captured by use of the ChemiDoc™ Touch Image System (Bio-Rad).

Subcellular Fractionation. Membrane, cytoplasmic and nuclear protein fractions were prepared using the Subcellular Protein Fractionation Kit for Cultured Cells (ThermoFisher) following the manufacture's protocol. Briefly, HEK293T cells with FNDC4 overexpression were collected and rinsed with cold PBS. Cell pellets were immersed in the first reagent which causes selective cytoplasmic membrane permeabilization, releasing soluble cytoplasmic contents. After collecting the cytoplasmic contents, a second reagent was added to dissolves cytoplasmic, endoplasmic, and mitochondria membranes but which did not solubilize nuclear membranes. After centrifugation, the intact nuclei were pelleted, and the supernatants were collected as was the membrane fraction. Nuclear pellets were used for soluble nuclear protein extraction. This protocol can also further separate soluble and chromatin-bound nuclear proteins, a step which was not performed in our study.

Protein Deglycosylation. The PNGase F protocol from New England Biolabs was used for the protein deglycosylation assay. Briefly, 10 μg of protein lysate from HEK293T cells with FNDC4 overexpression was denatured by heating at 100°C for 10 minutes. Denatured proteins were mixed with 1× GlycoBuffer 2 and 1% NP-40 to a total reaction volume of 20 μL. After adding 1 μL PNGase F, the reaction was incubated at 37°C for 1 hour. Results of deglycosylation were assessed by Western blot assay.

Co-IP and MS. The overexpression of FNDC4 proteins in NPCs were performed like that in the HEK293T cells but using the Lipofectamine™ Stem Transfection Reagent (ThermoFisher). Co-IP and MS were performed by following a protocol in our previous study (1) with minor modifications. Specifically, a Pierce™ c-Myc-Tag Magnetic IP/Co-IP Kit was used to pull-down overexpressed FNDC4 proteins which are fused with a MYC-tag at their C-termini. Western blot assay using anti-MYC antibody was performed to validate the pull-down efficiency. IP samples

were then applied to 10% Mini-PROTEAN® TGX™ Precast Protein Gel for protein separation. Protein bands were visualized by Coomassie blue staining and gel sections were cut for MS analysis. MS and the results analysis were done by the Taplin Biological Mass Spectrometry Facility at Harvard Medical School (Boston, MA).

***FNDC4* KO by CRISPR/Cas9**. The Alt-R™ CRISPR/Cas9 System (IDT) was used for *FNDC4* gene editing as described in our previous studies (2). This system includes a Hi-Fi Cas9 endonuclease, a gene-specific crRNA and a universal tracrRNA. These three components were combined to form a CRISPR/Cas9 ribonucleoprotein (RNP) complex and the RNP complex was delivered into cells for target DNA sequence cutting. Two crRNAs targeting *FNDC4* exon 3 and 4 DNA sequences (see Supplemental Table S1 for crRNA sequences), respectively, were designed for "double cuts" which would allow single-colony selection using standard PCR and agarose gel electrophoresis. The CRISPR/Cas9 RNP complex was delivered into iPSCs by electroporation using the P3 Primary Cell 4D-Nucleofector™ X Kit (Lonza) according to the manufacturer's instructions (Pulse code: CM-113). Two days after electroporation, edited iPSCs were passed to form single colonies. A portion of the cells (10%) was lysed with DNAzol® Direct and the lysates were used as PCR templates for the evaluation of CRISPR/Cas9 editing efficiency. The sequences of primers for amplification of the edited sites are listed in the Major Recourse table. The PCR was conducted with the KAPA HiFi HotStart ReadyMix PCR Kit (Roche). Once single colonies were formed, they were genotyped by PCR for the selection of homozygous KO colonies. Selected single-colony KO iPSCs were expanded for two more passages and were genotyped once again to confirm their KO status. Confirmed single-colony KO iPSCs were further characterized by karyotyping to ensure genomic integrity and by trilineage differentiation to ensure their pluripotency.

Generation of Forebrain Organoids from iPSCs. The STEMdiff™ Dorsal Forebrain Organoid Differentiation Kit (STEMCELL) was used to generate forebrain region-specific organoids by following the manufacture's protocol. That protocol can reliably and reproducibly generate forebrain organoids with cell types appropriate for the human cerebral cortex (3). Briefly, WT and *FNDC4* KO iPSCs were seeded in an AggreWell™800 24-well plate for uniform embryoid bodies (EBs) formation. After 6 days, the EBs were transferred to a 6-well ultra-low adherent plate (~30 EB aggregates per well) and were placed on a level shaker in the 37°C incubator for expansion until day 25. Expanded organoids were further cultured for ~20 days of forebrain organoid differentiation. Differentiated forebrain organoids were cultured in maintenance media until used. Specifically, three organoids differentiated from each iPSC lines were harvested at days 45, 90 and 150 for single nuclei isolation and fixation which were also used for snRNA-seq. On day 150, organoids were also harvested for cryosection and immunostaining.

Cryosection and Immunofluorescence (IF). Neural organoid cryosection and immunofluorescence were performed following the protocol entitled "Cryogenic Tissue Processing and Section Immunofluorescence of Neural Organoids" from STEMCELL. Specifically, forebrain organoids were fixed with 4% paraformaldehyde (PFA) overnight at 4℃. After fixation, organoids were equilibrated in 30% sucrose overnight at 4℃ and were then snap frozen and transferred to a -80°C freezer until used. Sectioning was performed in a Leica CM1520 cryostat at -26℃ with the section thickness set at 16 μm. Multiple serial sections were collected and mounted on glass slides. Sectioned slides were kept in a -20℃ freezer until used. Before immunostaining, sectioned slides were removed from the freezer and were allowed to dry at room temperature. Sections were then outlined with a Pap pen and were washed with PBST for 10 mins at 37°C to remove gelatin. Sections were blocked with 5% BSA/PBST for 1 hour, followed by

primary antibody incubation overnight at room temperature in a humidified chamber. After 3 washes with PBST, sections were incubated with fluorescence-labelled secondary antibodies at room temperature for 2 hours. After an additional 3 washes with PBST, slides were air dried at room temperature. Slides were then mounted with Mountant and were covered with coverslips. Stained slides were stored at 4°C before imaging using a Zeiss LSM 780 Confocal Microscope. See Supplemental Table S1 for antibody information.

Single Nuclei Isolation and Fixation. Single nuclei were isolated from fresh neural organoids harvested at days 45, 90 and 150 of differentiation/maturation. Specifically, three organoids generated from each iPSC line were transferred into a 15-mL tube and were rinsed with 1 mL of cold DPBS. After removing DPBS, chilled Lysis Buffer (Tris-HCl, 10 mM; NaCl 10nM; $MgCl_2$, 3mM; NP-40, 0.1%) was added and samples were incubated on ice for 30 mins to lyse the organoids. Organoids were then further triturated by pipetting 5-7 times to release nuclei. Nuclei were then pelleted by centrifugation at 500×g for 10min at 4°C. Nuclear pellets were resuspended in Nuclei Wash and Resuspension buffer (1% BSA and 0.2U/µL RNase inhibitor in PBS) and were mixed by gentle pipetting. Lysis efficiency was assessed by trypan blue staining with automated cell counting. If a high cell viability (>10%) remained, the lysis process was repeated until cell viability fell below 10%. Cell debris and large clumps were removed by using a 40 µm Flowmi Cell Strainer. Filtered single nuclei were washed twice using the Nuclei Wash and Resuspension buffer and were centrifuged at 500×g for 10 min at 4°C. Washed single nuclei were directly fixed using the Evercode™ Nuclei Fixation v2 kit (Parse Biosciences) by following the manufacture's protocol. Fixed and permeabilized nuclei were snap frozen and stored at -80°C until used.

snRNA-seq. Single-nucleus RNA samples were barcoded using the "split-pool" combinatorial barcoding technology (4). Specifically, frozen single nuclei were thawed on ice. Single nuclei

numbers and viability were analyzed using Nexcelom Cellometer Auto2000 with the AOPI fluorescent staining method. Single-nuclei library preparation was performed using the Evercode™ WT v2 kit (Parse Bioscience) according to the manufacturer's protocol. Approximately 16,000 fixed and permeabilized nuclei *per* sample were loaded into 48 wells of the Round 1 Plate. RNA was reverse transcribed using oligo dT and random hexamer primers with a well-specific barcode that was associated with specific samples. After 3 rounds of combinatorial barcoding, a total of ~150,000 nuclei from 9 samples were recovered. Barcoded nuclei were then split into 8-sublibrary tubes (Round 4) and were lysed (Figure 3A). After nuclear lysis, cDNA was captured, amplified, and quantified by Qubit DNA HS assay kit. The multiplexed sublibraries were pooled and sequenced on a Novaseq X Plus, 10B flowcell using paired-end 150nt (PE150) mode. Library preparation and sequencing were performed at the Northwestern University NUSeq core.

snRNA-seq Data Analysis. The raw sequencing FASTQ data were processed using the Parse Bioscience split-pipe pipeline (v1.4.0) with default settings to align sequencing reads to the human genome (hg38) and to demultiplex samples. Downstream analysis was performed in R (v 4.3.2), with data filtering and initial analyses performed using Seurat (v.5.0.3) (5). Genes were filtered to remove those expressing in less than 100 nuclei, and nuclei were filtered to only include those with at least 500 genes detected. A set of 2000 genes with the highest variance across all cells were then chosen as variable features. All 9 single-nuclei forebrain organoid samples (3 iPSC lines × 3 time points) were then integrated together, and anchor genes were identified for further unsupervised clustering analyses. Clustering of single nuclei was performed using the Uniform Manifold Approximation and Projection (UMAP) (6), identifying 15 distinct single nuclei clusters with a resolution of 0.5. Clusters were then labeled for distinct brain cell types using ScType (7) with prior neural organoid datasets (8). Cluster marker genes were determined using the Seurat

function FindAllMarkers for each cluster and filtered by Bonferroni-corrected p value < 0.05 and |log2 fold change| > 1. We compared the difference between conditions by looking at the clustering differences, differential gene expression between samples, as well as differential gene expression between clusters of the same samples. When comparisons between samples were made, random down-sampling was performed using the "sample()" function on the larger sample to allow for equal numbers of nuclei/cells for major comparisons, and multiple down-samples were used.

Because these forebrain organoids included cells at various differentiation and maturation stages, trajectory and pseudotime analyses were performed using the Monocle3 package (v. 1.3.7) (9), with trajectories calculated independently between samples. For all samples pooled, a resolution of 3e-5 was used, while for WT alone, KOc2 alone, and KOc5 alone (all including both d45 and d90), a resolution of 3e-3 was used. A root cluster was set in the Neural Progenitor Cell cluster(s) for all samples from which pseudotime and trajectories could branch.

MEA Assay. We randomly picked one KO iPSC line (SC#2), together with the WT iPSC, to generate dorsal forebrain organoids for the assay. Organoids at day 90 were plated onto CytoView 24-well plates (Axion Biosystems) coated with 0.1% polyethyleneimine (PEI) and 10 ug/ml laminin. The organoids were maintained in forebrain organoid maintenance medium for 3 days and were then switched to BrainPhys™ Neuronal Medium with half-medium changes every 3 to 4 days until day 120. Before MEA recording, the media was changed to the "recording media" (BrainPhys™ Neuronal Medium including 2% NeuroCult SM1 Neuronal Supplement, 1% N2 Supplement-A, 1mM Dibutyryl-cAMP, 200 nM Ascorbic Acid, 10 mM [+] D-glucose solution, 20ng/mL BDNF, and 20ng/mL GDNF), and organoids were equilibrated in "recording media" for 1.5 hours until first (baseline) MEA recording (see Figure 8A). Spontaneous electrical activity was recorded at 37 °C using the Neural + Viability Real-Time configuration (Maestro Axis Software,

version 3.12.10, Axion Biosystems). The plates were left to adjust in the Maestro Edge for 5 minutes prior to recording and were then recorded continuously for up to 3 minutes. An electrode was considered active if it met the minimum spike rate threshold of 5 spikes per minute. Each well of the MEA plate has 16 electrodes. After MEA recording, active electrodes from each well were automatically counted. A well/organoid with less than 6 active electrodes in the MEA assay were removed from the final analysis. Burst was defined using the Inter-Spike Interval Threshold, with at least 5 spikes on an electrode, each separated by an inter-spike interval of no more than 100 milliseconds. Network burst was defined using envelope threshold, with a threshold factor of 1.5, minimum inter-burst interval of 100 milliseconds, minimum electrodes of 35%, and burst inclusion of 75%. Synchrony window was defined as 20 milliseconds. Multielectrode data analysis was performed using the Axion Biosystems Neural Metrics Tool version 4.1.3.

References:

1. Liu D, Zhuang Y, Zhang L, Gao H, Neavin D, Carrillo-Roa T, et al. ERICH3: vesicular association and antidepressant treatment response. *Mol Psychiatry.* 2021;26(6):2415-28.
2. Nguyen TTL, Gao H, Liu D, Philips TJ, Ye Z, Lee JH, et al. Glucocorticoids unmask silent non-coding genetic risk variants for common diseases. *Nucleic Acids Res.* 2022;50(20):11635-53.
3. Yoon SJ, Elahi LS, Pasca AM, Marton RM, Gordon A, Revah O, et al. Reliability of human cortical organoid generation. *Nat Methods.* 2019;16(1):75-8.
4. Rosenberg AB, Roco CM, Muscat RA, Kuchina A, Sample P, Yao Z, et al. Single-cell profiling of the developing mouse brain and spinal cord with split-pool barcoding. *Science.* 2018;360(6385):176-82.
5. Hao Y, Stuart T, Kowalski MH, Choudhary S, Hoffman P, Hartman A, et al. Dictionary learning for integrative, multimodal and scalable single-cell analysis. *Nat Biotechnol.* 2024;42(2):293-304.
6. Becht E, McInnes L, Healy J, Dutertre CA, Kwok IWH, Ng LG, et al. Dimensionality reduction for visualizing single-cell data using UMAP. *Nat Biotechnol.* 2018.
7. Ianevski A, Giri AK, and Aittokallio T. Fully-automated and ultra-fast cell-type identification using specific marker combinations from single-cell transcriptomic data. *Nat Commun.* 2022;13(1):1246.
8. Dang J, Tiwari SK, Agrawal K, Hui H, Qin Y, and Rana TM. Glial cell diversity and methamphetamine-induced neuroinflammation in human cerebral organoids. *Mol Psychiatry.* 2021;26(4):1194-207.
9. Cao J, Spielmann M, Qiu X, Huang X, Ibrahim DM, Hill AJ, et al. The single-cell transcriptional landscape of mammalian organogenesis. *Nature.* 2019;566(7745):496-502.

Supplemental Figures S1-S12

Figure S1. The rs56951679 SNP (chr2:28260851; T>C) is an sQTL for FNDC4 in multiple human brain regions (Cortex, Nucleus Accumbens, Frontal Cortex, Hippocampus and Caudate) based on the RNA-seq data generated by GTEx. The variant allele, “C”, is associated with an increased level of intron excision at chromosome 2: 27,492,478-27,493,389 (human genome assembly build 38).

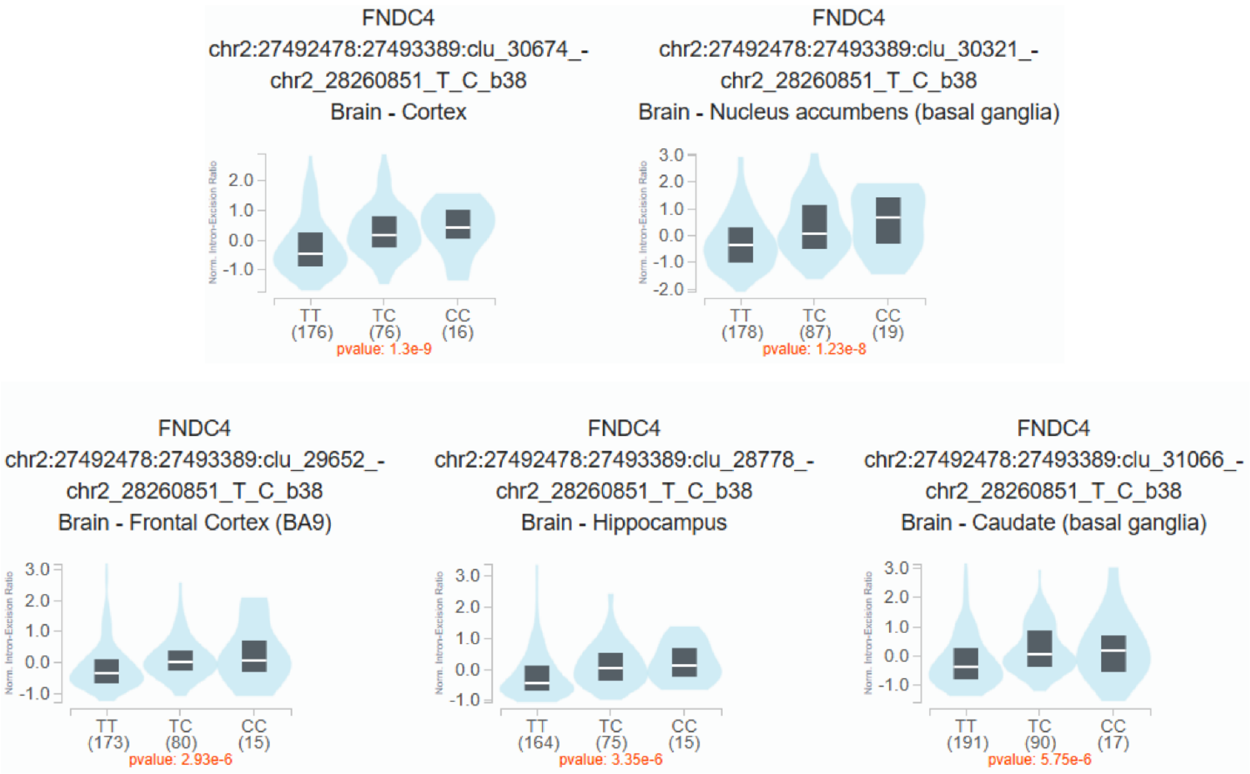

Figure S2. The rs1260326 SNP (chr2:27508073; C>T), which has been associated with AUD and alcohol consumption in GWAS, is an sQTL for FNDC4 in multiple human brain regions based on the RNA-seq data generated by GTEx. The common allele "C" is associated with an increased level of intron excision at chromosome 2: 27,492,478-27,493,389 (human genome assembly build 38).

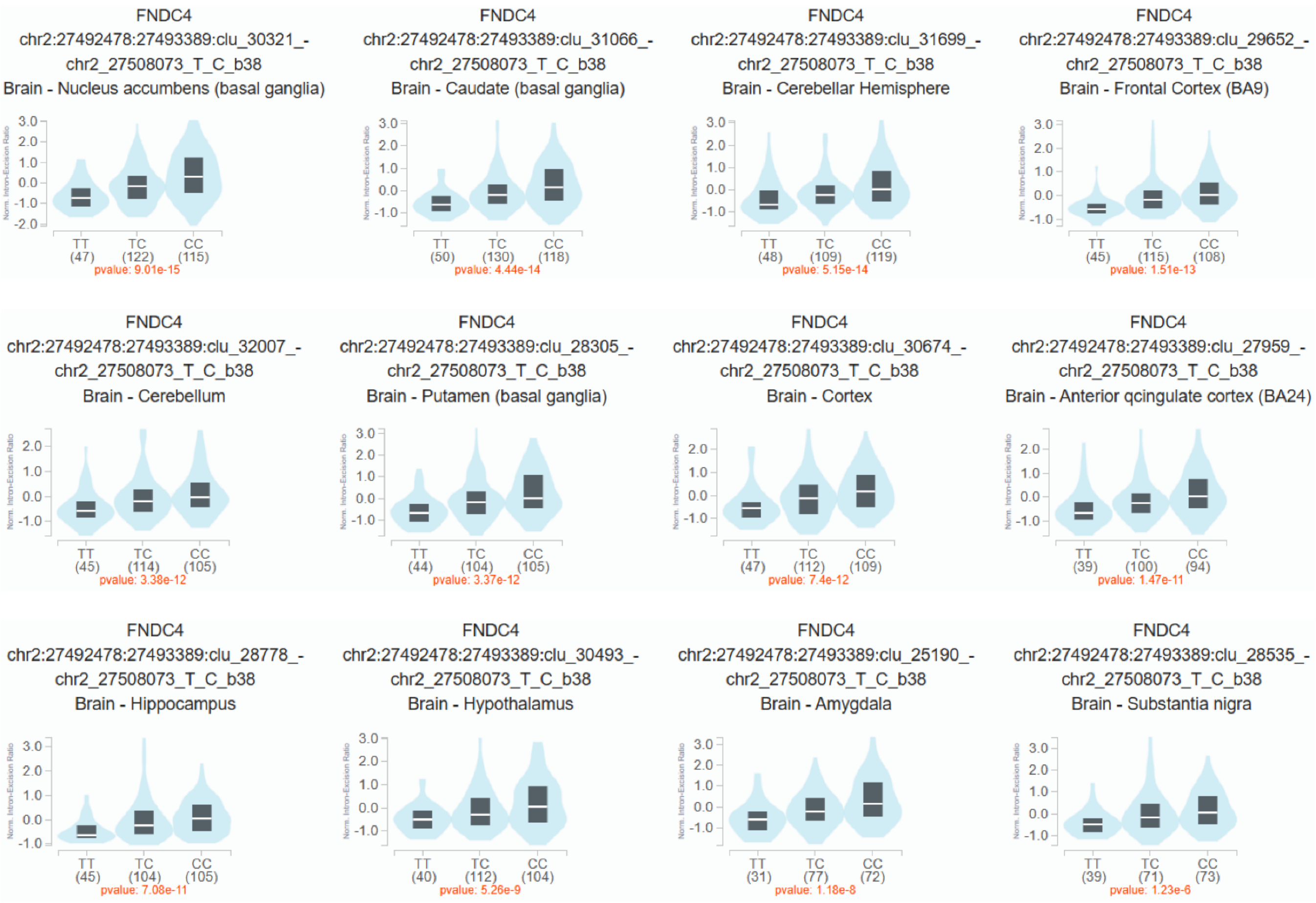

Figure S3. (**A**) The Western blot shows cDNA-overexpressed canonical and truncated FNDC4 proteins (fused with MYC- and FLAG-tags at their C-termini) using anti-MYC antibodies. Beta-actin (ACTB) was blotted as a loading control. (**B**) Those same protein samples were then used to test anti-FNDC4 antibodies. Five of those tested FNDC4 antibodies detected overexpressed FNDC4 proteins, but they varied in efficiency and specificity. (**C**) Table shows the basic information of 5 tested FNDC4 antibodies (see Table S1 for details). Antigen aa number was based on the aa sequences of canonical FNDC4. (**D**) Western blots for endogenous FNDC4 presents in protein lysates of human whole brain (WB), cerebral cortex (CC) and cerebellum (CB). Overexpressed canonical and truncated FNDC4 proteins were blotted as positive controls. Protein marker (M) was loaded to separate human brain protein lysates from overexpressed FNDC4 proteins. (**E**) Detection of FNDC4 proteins in culture media for HEK293T cells overexpressing FNDC4 proteins (fused with MYC- and FLAG-tags at their C-termini) using anti-MYC-tag antibody. Culture media collected in three overexpression experiments (Exp) were concentrated (20 ×) and tested with cell lysate in a same blot. Protein marker (M) was loaded to separate cell lysate and culture media samples.

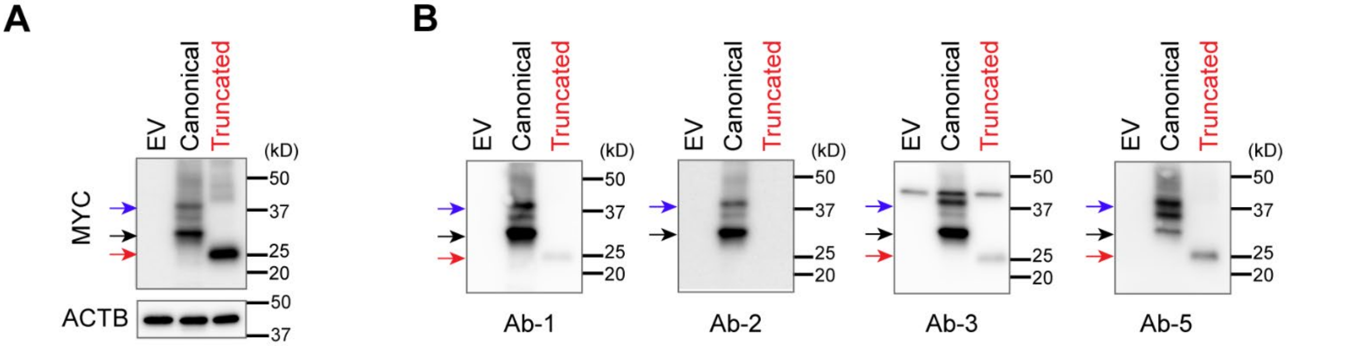

C

FNDC4 Ab#	Clone ID	Antigen amino acids (aa)
Ab-1	OTI3B11	aa1 – aa234
Ab-2	N/A	aa194 – aa234
Ab-3	OTI1B7	aa1 – aa234
**Ab-4**	**OTI3E10**	aa1 – aa234
Ab-5	7F9	aa45 – aa154

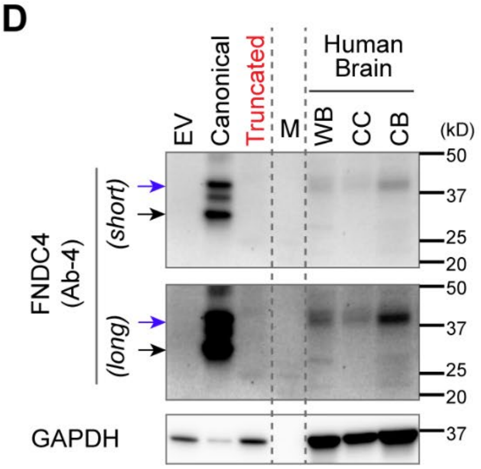

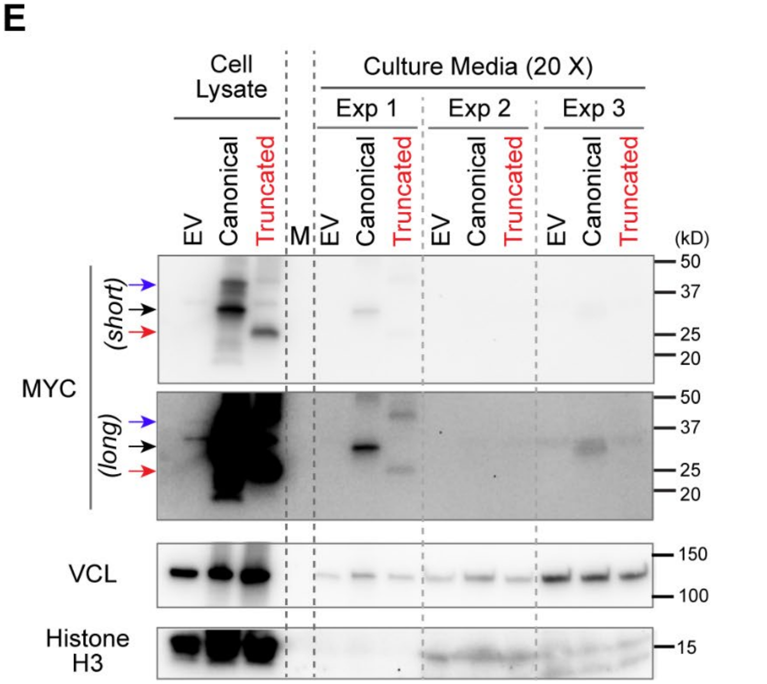

Figure S4. Amino acid sequences alignment for human, rat and mouse FNDC4 proteins. Signal peptide (SP) sequences were highlighted in red. Alignment was performed using the protein sequence data in the UniProtKB.

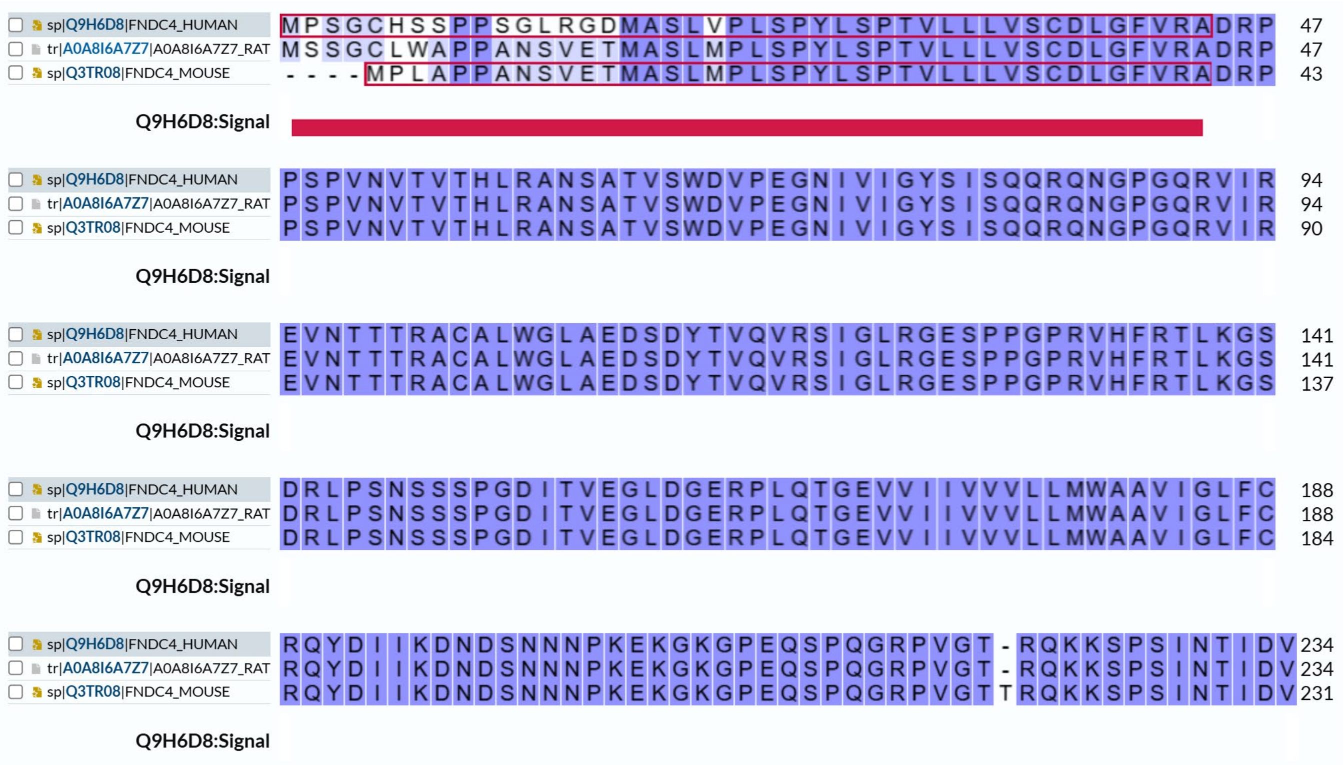

Figure S5. FNDC4-interacting proteins identified by the biophysical interactions of ORFeome-based complexes (BioPlex) network, a project identifies proteome-scale protein interaction networks through affinity-purification mass spectrometry (22). Figure was obtained from the BioPlex Exloper (https://bioplex.hms.harvard.edu/explorer/). Accessed on November 15th, 2025.

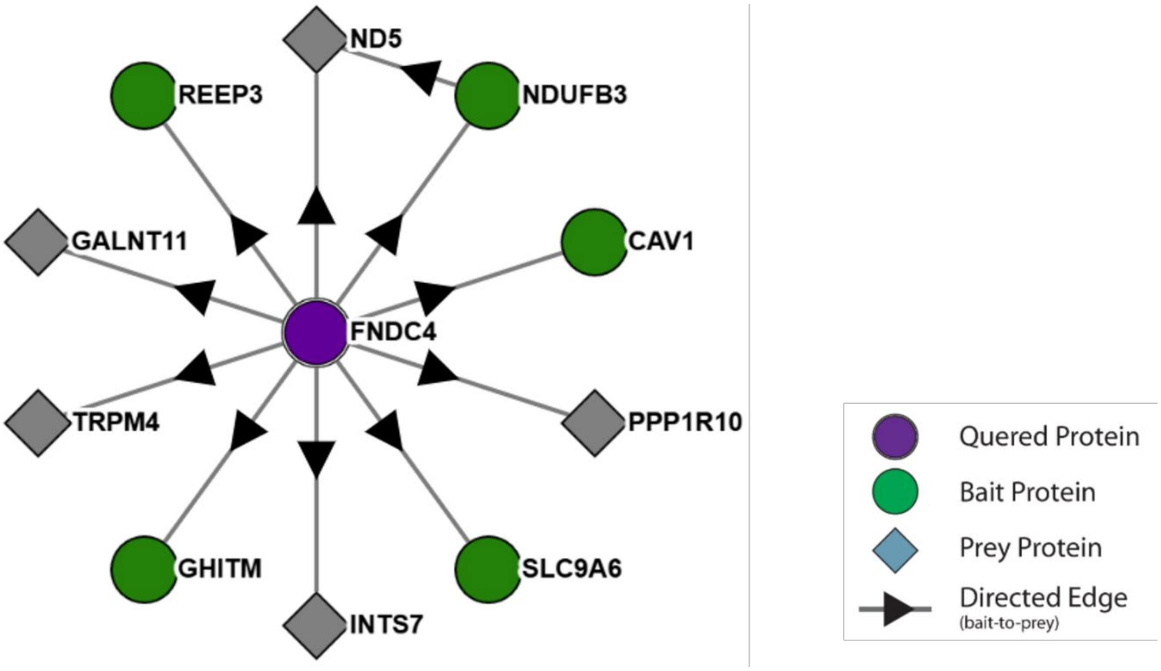

Figure S6. Pathways/terms enrichment of (**A**) Gene Ontology (GO) Molecular Function and (**B**) COMPARTMENT using the 242 canonical FNDC4-interacting proteins identified in human iPSC-derived NPCs. Extracellular matrix and membrane-related terms are highlighted in red and purple, respectively. Although with much less significant *p*-value than that of cellular-membranes-related terms, protein translation- and degradation-related terms (such as "mRNA Processing/Binding", "Ubiquitin Protein Ligase Binding") were also enriched. However, we believed these enriched terms were due to the pull-down of proteins that are involved in processing of overexpressed FNDC4 but not proteins that are functioning with FNDC4, thus might not inform the FNDC4 function.

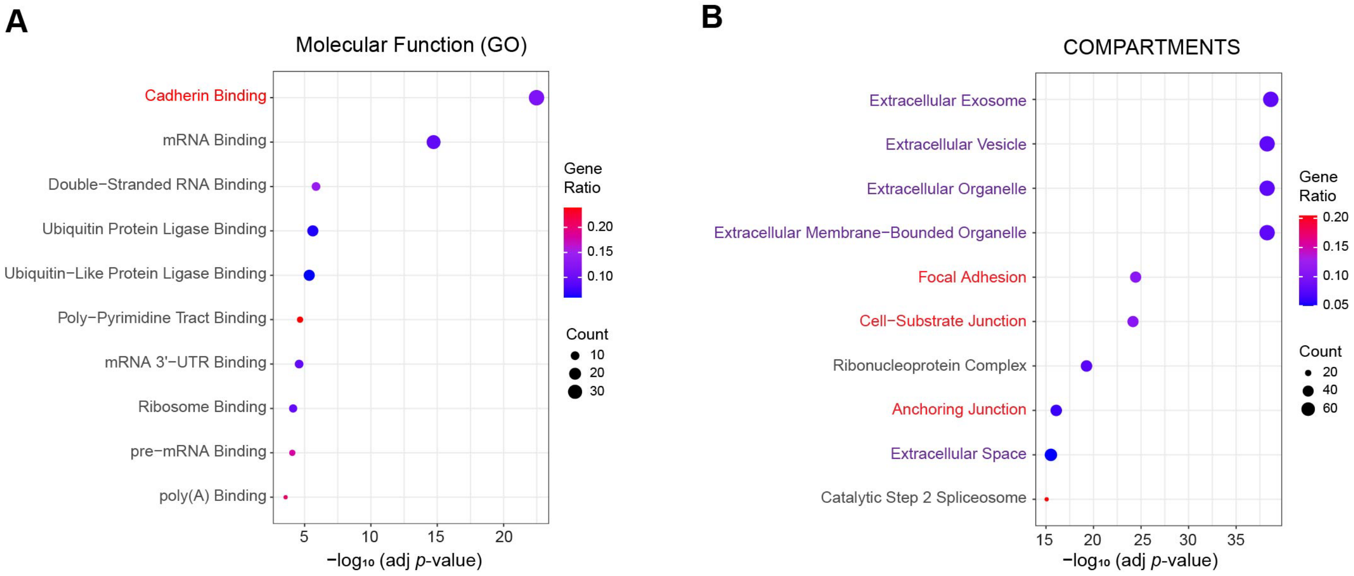

Figure S7. Generation and Characterization of *FNDC4* KO iPSCs. (**A**) Scheme for the generation of *FNDC4* KO iPSC lines using CRISPR/cas9 gene editing. (**B**) Design of guide RNAs (gRNAs) for *FNDC4* KO, and of PCR primers for KO colony selection. Successful CRISPR/cas9 editing by both gRNAs has been designed to remove 388 base pairs (bp) from the *FNDC4* gene, resulting in a 554 bp amplicon by PCR using designed primers. (**C**) Agarose gel results showing the PCR amplicons when genomic DNAs from the WT control and CRISPR/cas9-edited iPSCs (mixed) were used as PCR templates. The presence of a smaller band (~554 bp) in the edited iPSCs (lanes 1 and 2) indicates successful *FNDC4* KO in certain individual cells. Lanes 1 and 2 are the same CRISPR/cas9 experiments with slight differences in transfection methods to deliver CRISPR/cas9 RNP complexes. These "mixed" *FNDC4* KO iPSCs were used for single-colony isolation. (**D**) Selection of single-colony *FNDC4* KO iPSCs by PCR. Genomic DNA extracted from single-colony iPSC lines was used as PCR templates and the PCR amplicons were visualized in agarose gel. Four single-colony iPSC lines (colony numbers highlighted in red) which are potentially homozygous *FNDC4* KO were passed and populated for further validation. (**E**) After 3 passages of the four *FNDC4* KO iPSC lines, they were genotyped once again by PCR to validate the homozygosity of *FNDC4* KO. Colonies A2 and A5 (renamed as colonies #2 and #5, respectively), which maintained the homozygosity of *FNDC4* KO, were used in further studies. A section of this picture without showing colonies D2 and D3 is presented as Figure 4C. (**F**) The WT and both homozygous *FNDC4* KO iPSC lines had their genomic integrity confirmed by karyotyping. Karyotypes for both *FNDC4* KO iPSC lines are also presented as Figure 4D.

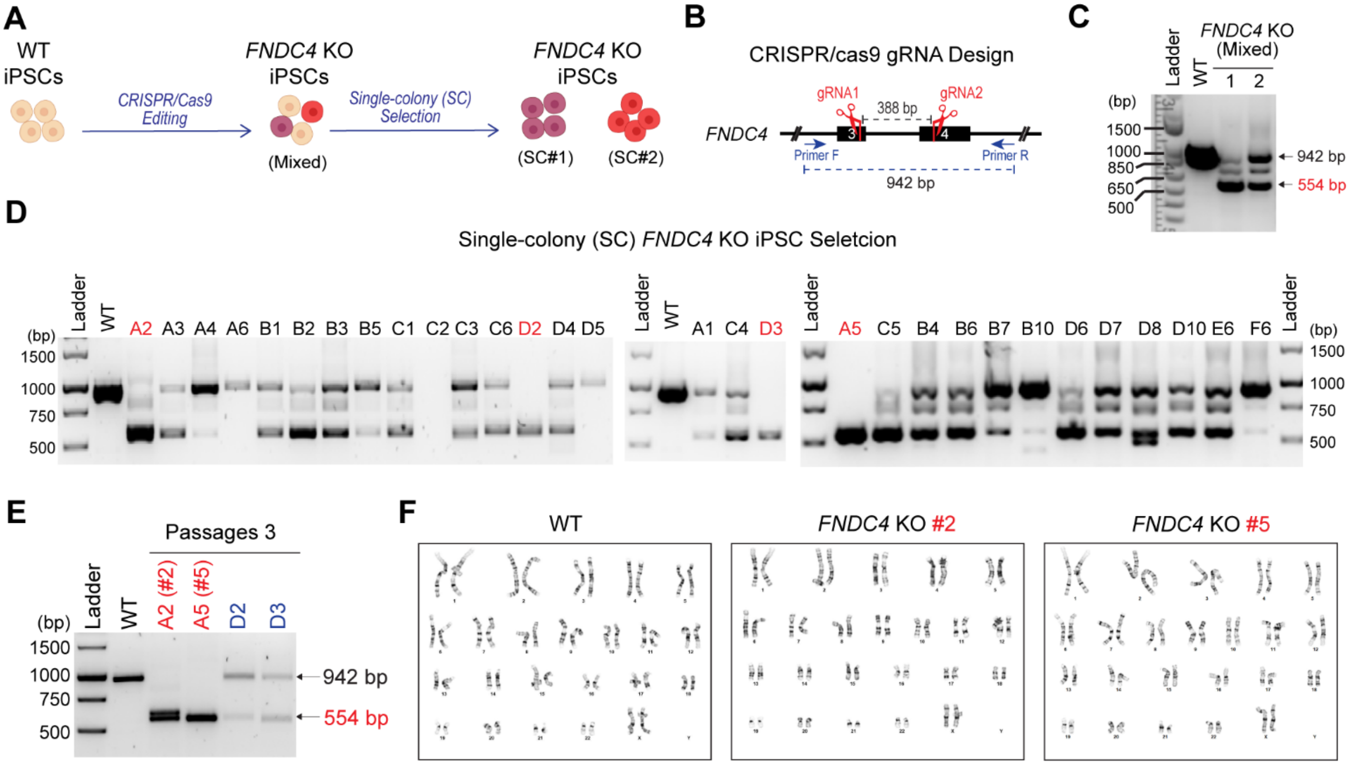

Figure S8. Generation of forebrain organoids from human iPSCs. (**A**) Scheme for forebrain organoid generation. Human iPSCs were seeded in AggreWell™800 to form embryoid bodies (EBs). After 6 days, EBs were transferred to a suspension culture plate for organoid expansion until day 25. Organoids were then cultured in forebrain organoid differentiation medium. After approximately 3 weeks of differentiation, organoids were switched to maintenance media and cultured for up to 150 days. Three organoids from each iPSC line (WT, *FNDC4* KO#2 or KO#5) were harvested at three time points (d45, d90 and d150) for single nuclei isolation and snRNA-seq. Forebrain organoids at day 150 were cryosectioned and characterized by immunofluorescence (IF) staining of neural markers. (**B**) Forebrain organoids in differentiation. Pictures were taken under a microscope with 10× magnification. (**C**) Forebrain organoids in maintenance. Pictures were taken by iPhone.

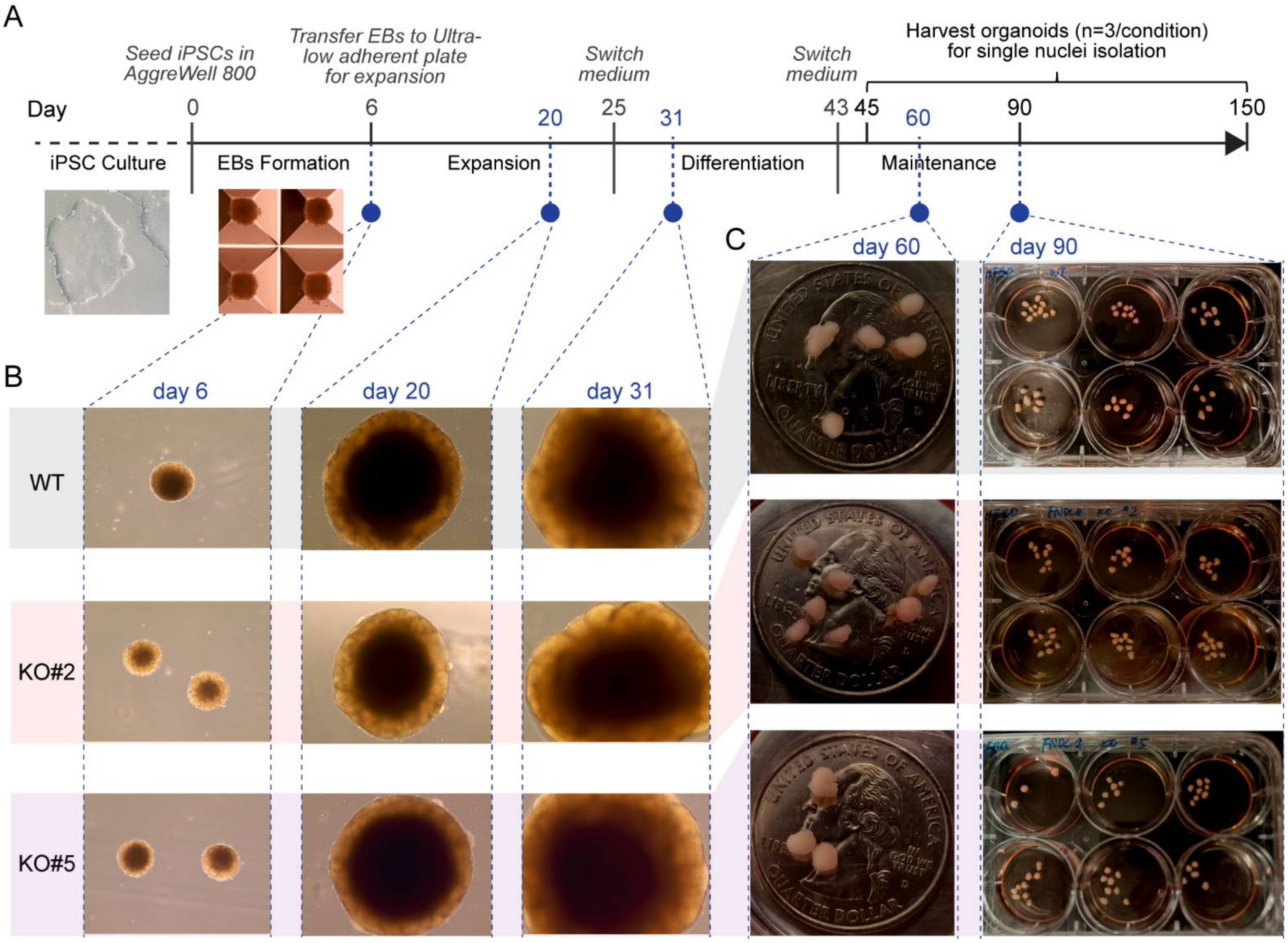

Figure S9. Pie charts showing the proportion of single nuclei for each annotated cell type in (**A**) total sequenced single nuclei (n=103,263), and in random down-sampling single nuclei samples under (**B**) 9 or (**C**) 6 experimental conditions. Random down-sampling was performed to ensure that each experimental condition would include a same number of single nuclei for comparison of cell type proportions. That same number was determined by single-nuclei number of the condition which had the least available single nuclei. (**D**) Pie charts showing the proportions of annotated cell types in dorsal forebrain organoids for 6 individual experimental conditions (WT, KO#2 and KO#5 at day 45, and 90 of organoid differentiation/maturation). When pie charts for individual conditions were compared, increases in the proportion of GluN (green) were observed in *FNDC4* KO organoids. (**E**) These single nuclei from 6 individual experimental conditions were also visualized by UMAP plots. (**F**) Violin plots comparing proportions of eight cell types in WT and two *FNDC4* KO organoids at days 45 (d45) and 90 (d90) after three-rounds of random down-sampling. *P* values were calculated by two-way ANOVA with Dunnett's multiple comparisons to WT samples. **P* <0.05, ****P* <0.001, ns = not significant. *P* values for cell types with a proportion of 5% (dashed line) or less were not shown.

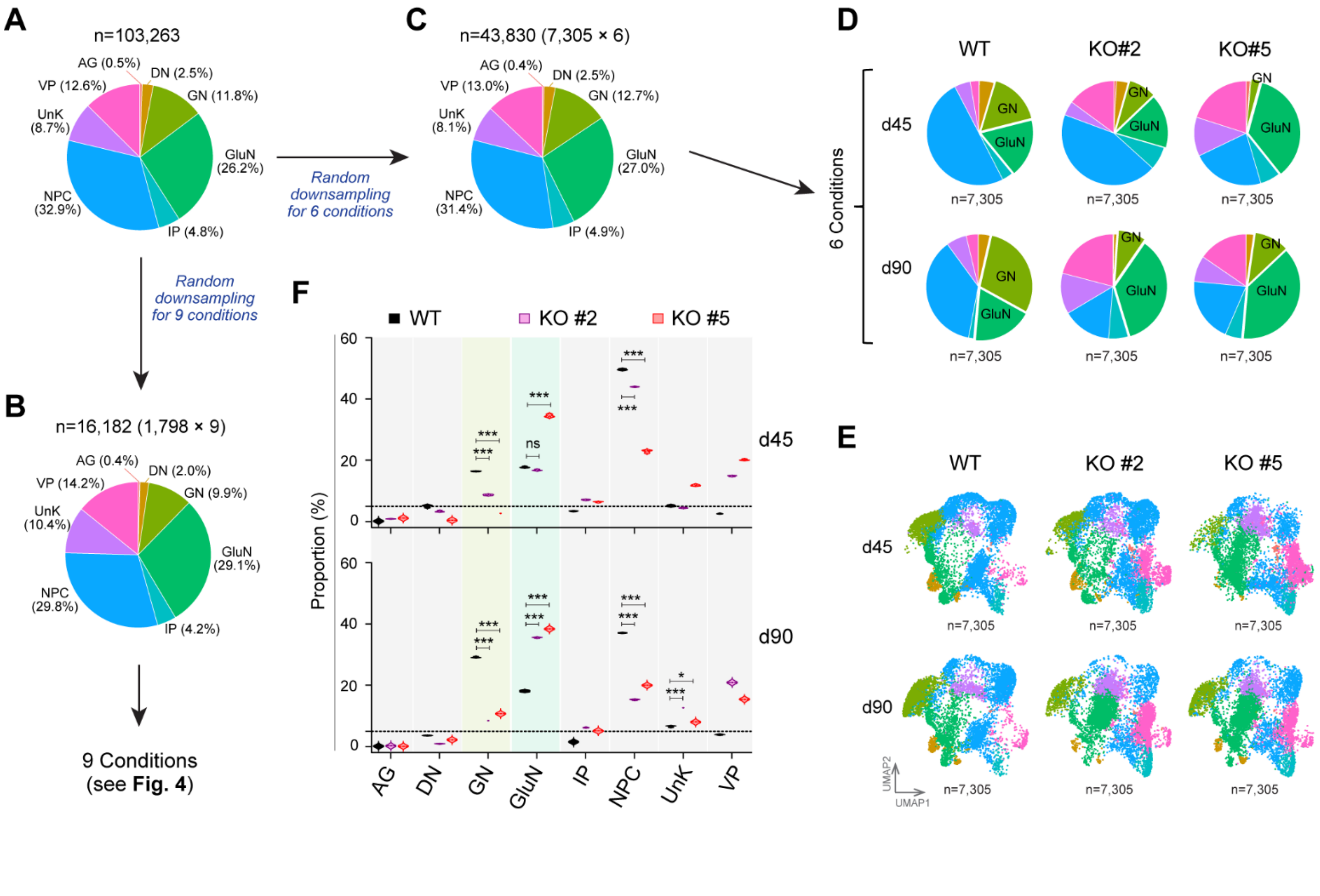

Figure S10. Gene ontology (GO) enrichment for (**A**) Cellular Compartment and (**B**) Molecular Function using the 221 differentially expressed genes in UnK cells from the *FNDC4* KO to WT forebrain organoids.

A

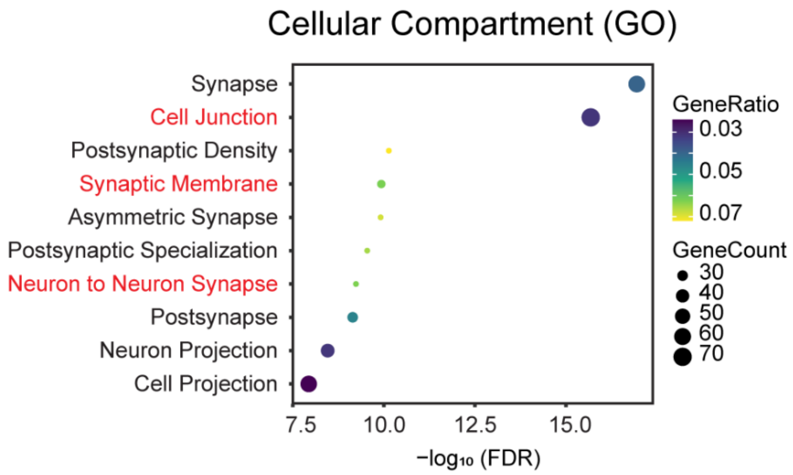

B

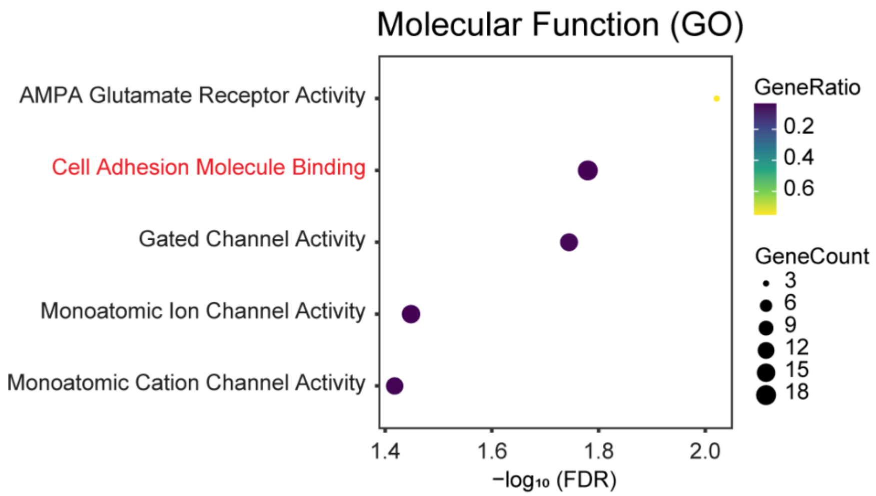

Figure S11. Scatter plots show the baseline (**A**) activity metrics, including number of total spikes, weighted mean firing rate (WMFR) and number of active electrodes, and (**B**) two network burst metrics (burst peak and synchrony index) of those WT and *FNDC4* KO forebrain organoids (n=10/group) within 3 minutes of continuous MEA recording. Each dot/square represents a single organoid. Bars represent mean values with error bar indicates SD. NS = not significant, ***indicates *p* <0.001, ****p <0.0001 in the Mann-Whitney test.

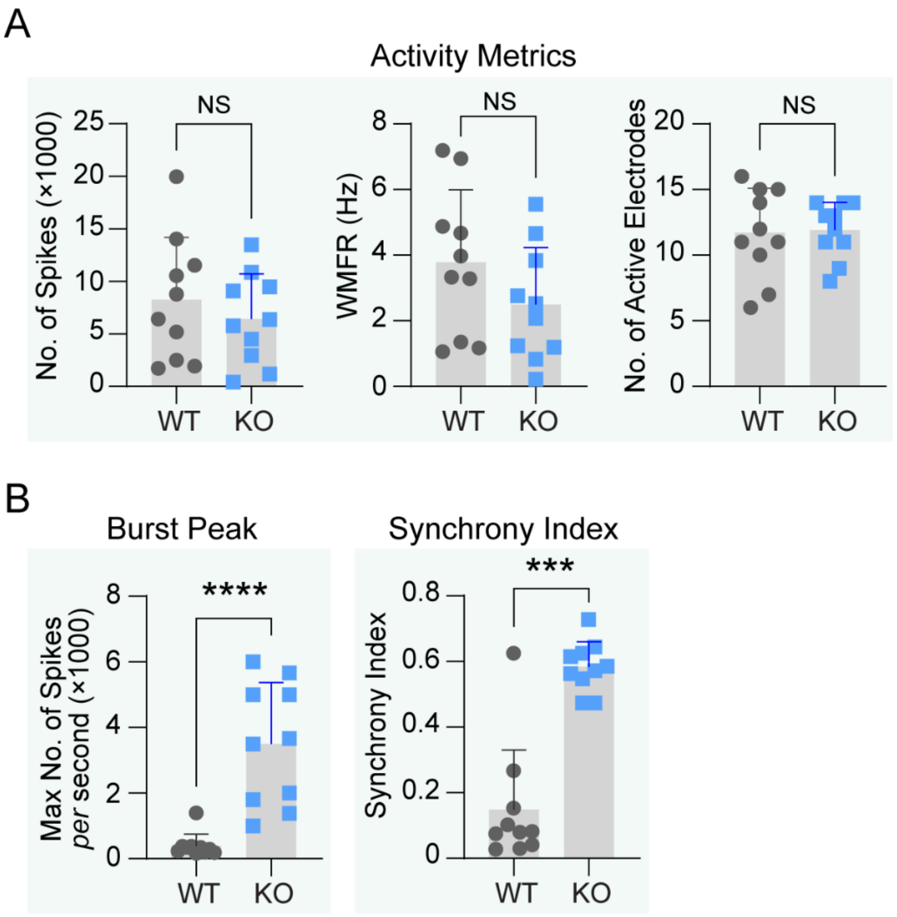

Figure S12. (**A**) Optimization of neural organoid MEA assay with ethanol (EtOH) exposure. Plot showing the $\log_2$ fold changes (FC) in total number of spikes after individual organoids were exposed to 0, 6.25, 12.5 and 25 mM of ethanol (EtOH). Data was collected at every hour of EtOH exposure, for up to 12 hours. (**B**) $\mathrm{Log_2}$FC in weighted mean firing rate (WMFR) and average burst duration in WT and *FNDC4* KO organoids (n=8/group) after 0.5, 1, 1.5 and 2 hours of EtOH (12.5 mM) exposure. Dots/squares represent mean value with error bars indicate SD. No significant difference between WT and KO groups was observed.

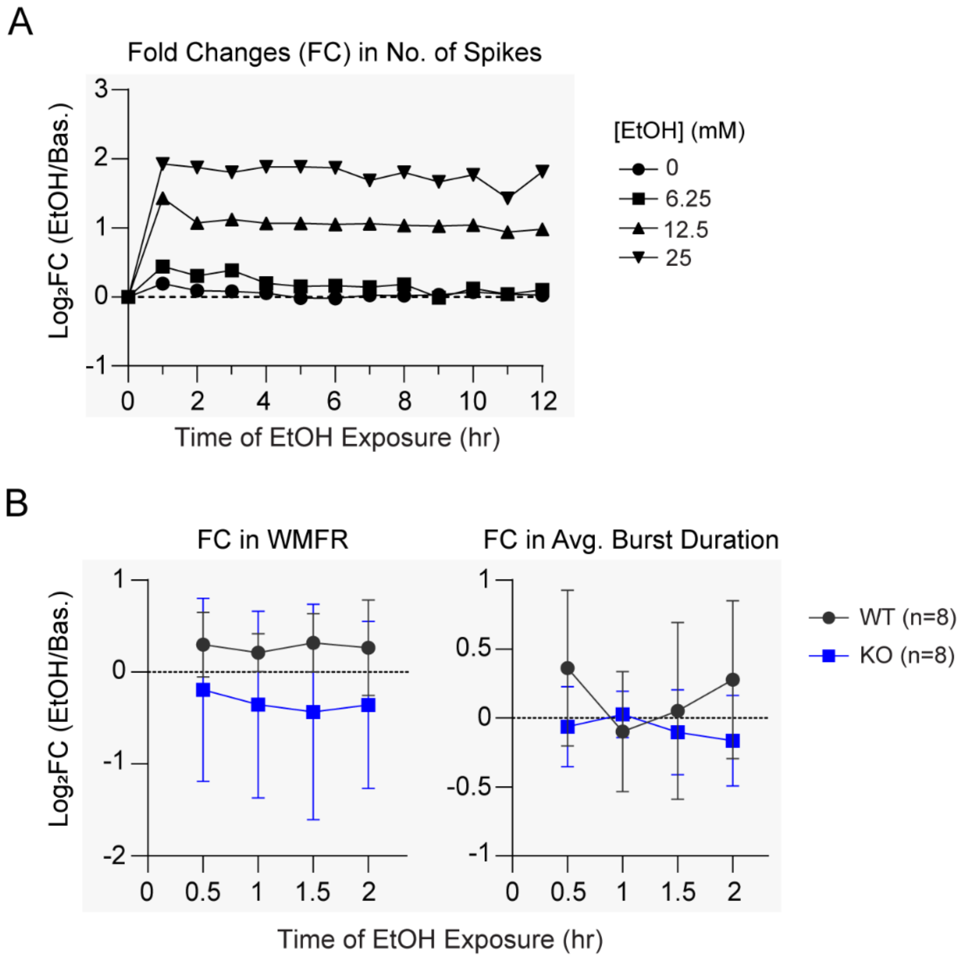

Supplement: Supplemental data [file jci-136-193204-s078.pdf]
