# Supplementary material for: Alcohol use disorder–associated gene FNDC4 alters glutamatergic and GABAergic neurogenesis in neural organoids
Source: J Clin Invest. 2026 Jan 8;136(5):e193204. doi: 10.1172/JCI193204 (PMC12948423; doi:10.1172/JCI193204)

Original Western Blots (pre-cut)

Alcohol Use Disorder Associated Gene *FNDC4* Alters Glutamatergic and GABAergic Neurogenesis

Zhu X, *et al*. 2025

Introduction

1. All Western blot pictures were obtained by using a Bio-Rad ChemiDoc Touch Imaging System.
2. A Bio-Rad Precision Plus Protein Dual Color Standards (cat#:1610374, see image for molecular weights of markers) was used for all blots in this study.
3. On a single blot (PVDF membrane), **protein bands** were visualized by Chemiluminescence, and **prestained protein markers (standards)** were visualized by Colormetric picture.
4. Chemiluminescence and Colormetric pictures were **Merged** to show the sizes of bands (see examples below).
5. All pre-cut blots shown in subsequent slides are Merged pictures with **red-dashed boxes** to indicate cut blots that are presented in the Figures. (see pictures below for examples: red-dashed box corresponding to **Figure 2B**)

Bio-Rad
(cat#:1610374)

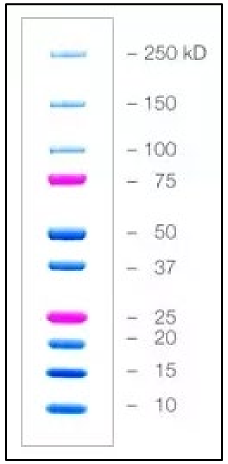

Protein Blot
(Chemiluminescence)

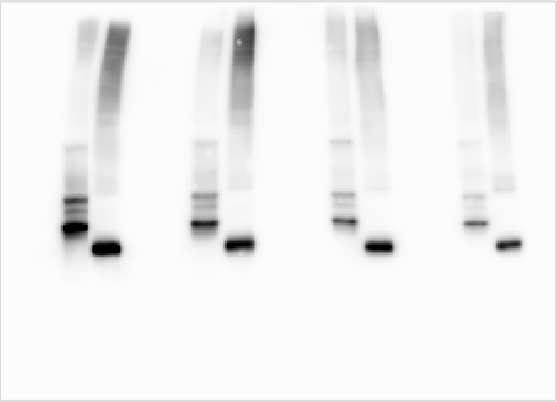

+

Prestained Markers
(Colormetric)

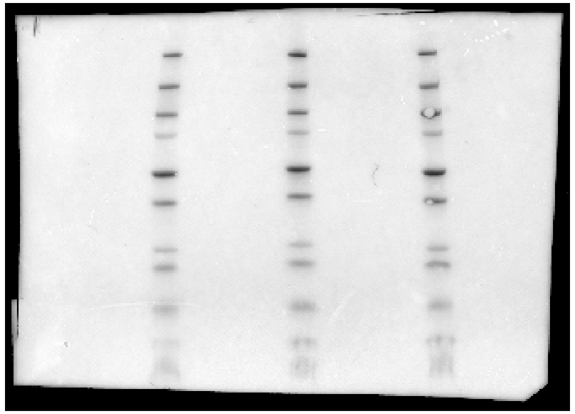

=

Merged

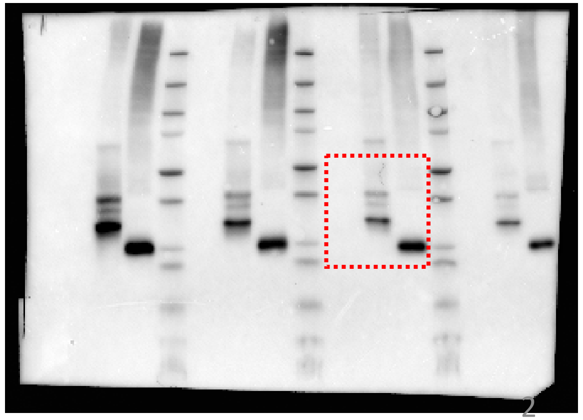

Full unedited blot for **Figure 2B** & **Supplemental Figure S3A**

Figure 2B

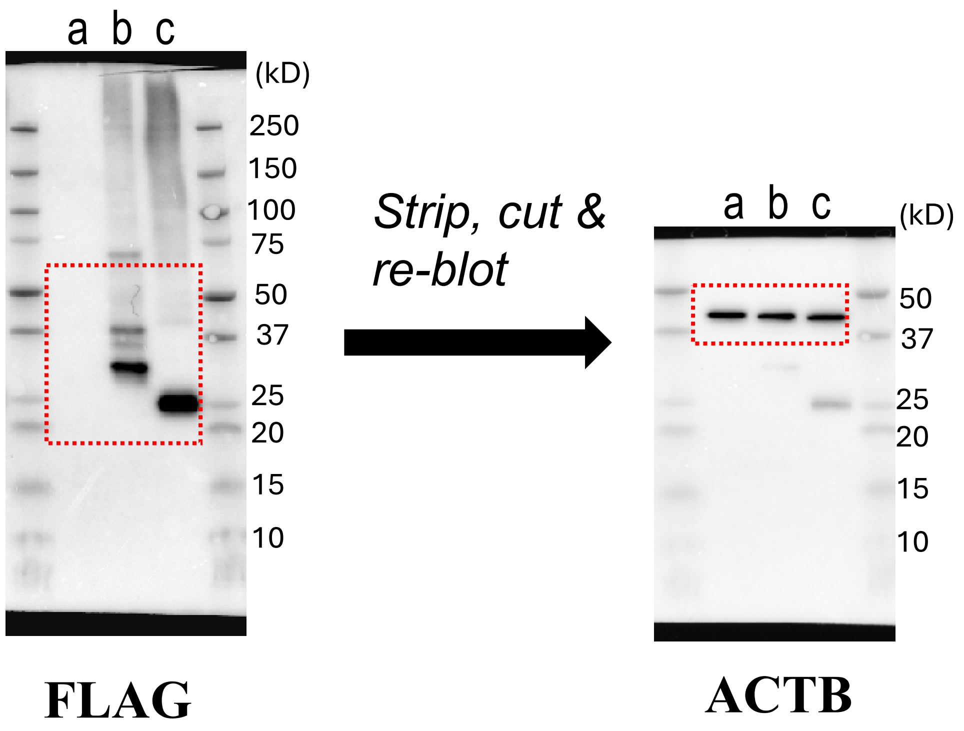

Figure S3A

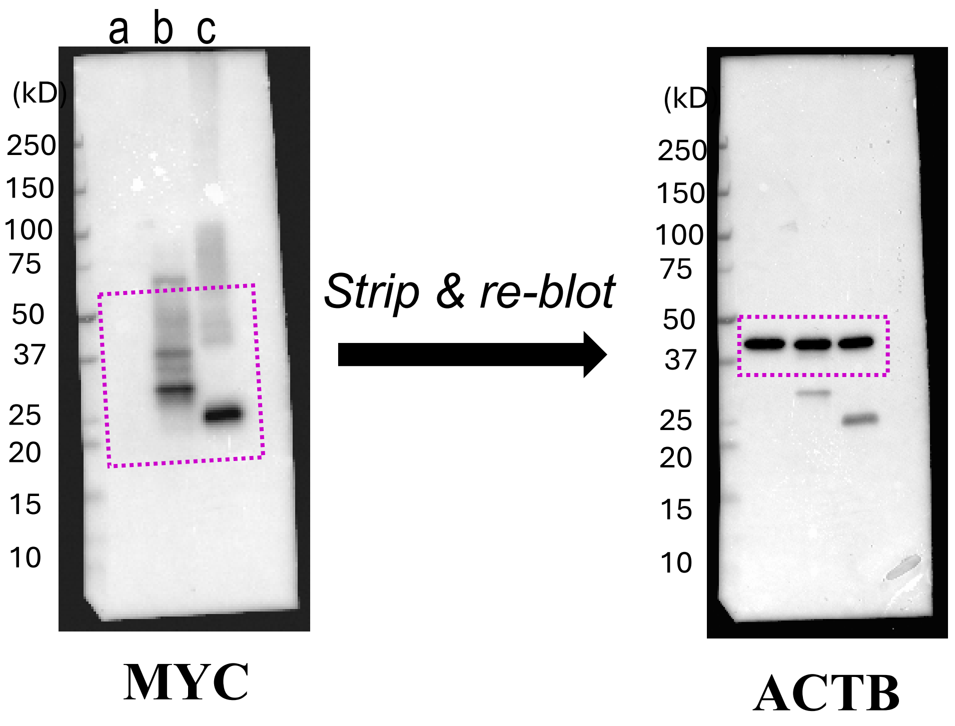

Sample info:

a: Empty vector (EV)
b: Canonical FNDC4 over-expression (MYC-FLAG-tagged)
c: Truncated FNDC4 over-expression (MYC-FLAG-tagged)

Full unedited blot for Figure 2C & Supplemental Figure S3B

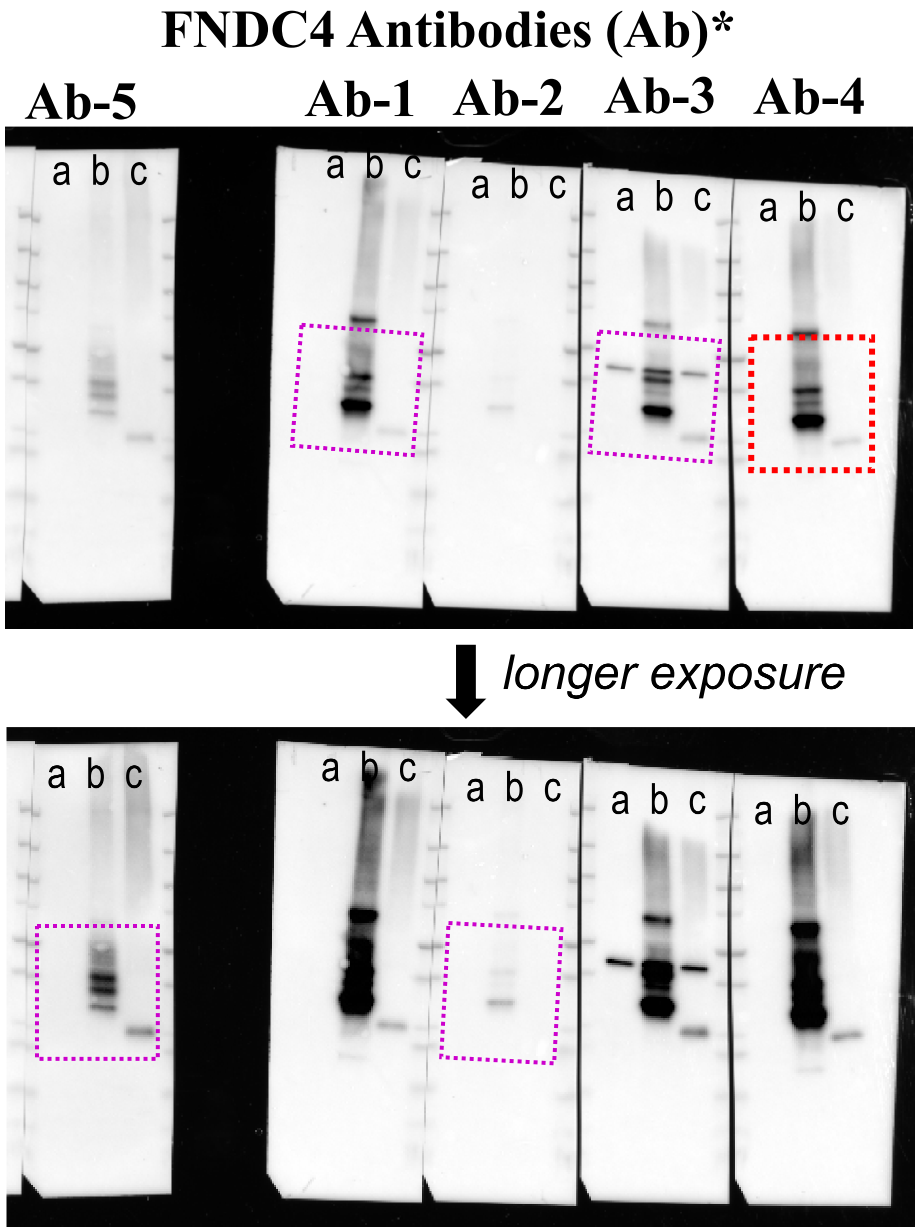

Strip & re-blot

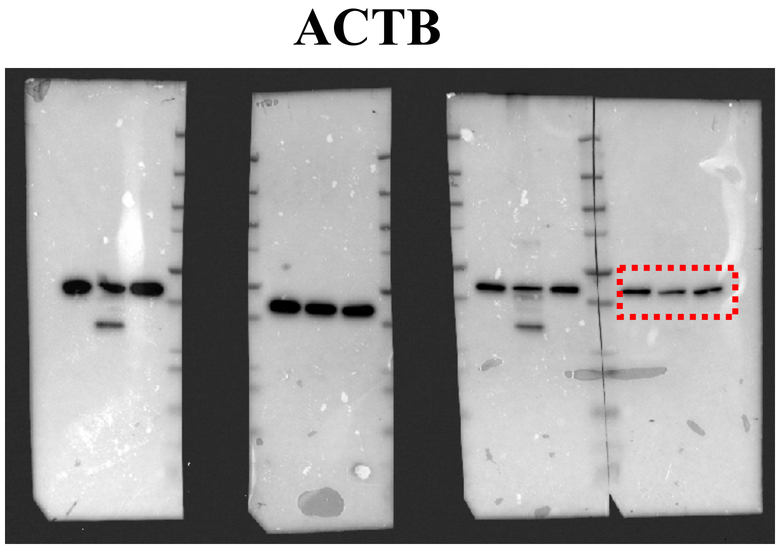

*** FNDC4 Ab info:**

Ab#	Source	Catalog number	Clone ID	Antigen amino acids (aa)
Ab-1	OriGene	TA505462	OTI3B11	aa1 – aa234
Ab-2	ThermoFisher	PA5-62486	N/A	aa194 – aa234
Ab-3	OriGene	TA505463S	OTI1B7	aa1 – aa234
**Ab-4**	**OriGene**	**TA505459**	**OTI3E10**	aa1 – aa234
Ab-5	Abnova	H00064838-M01	7F9	aa45 – aa154

Full unedited blot for **Figure** 2D

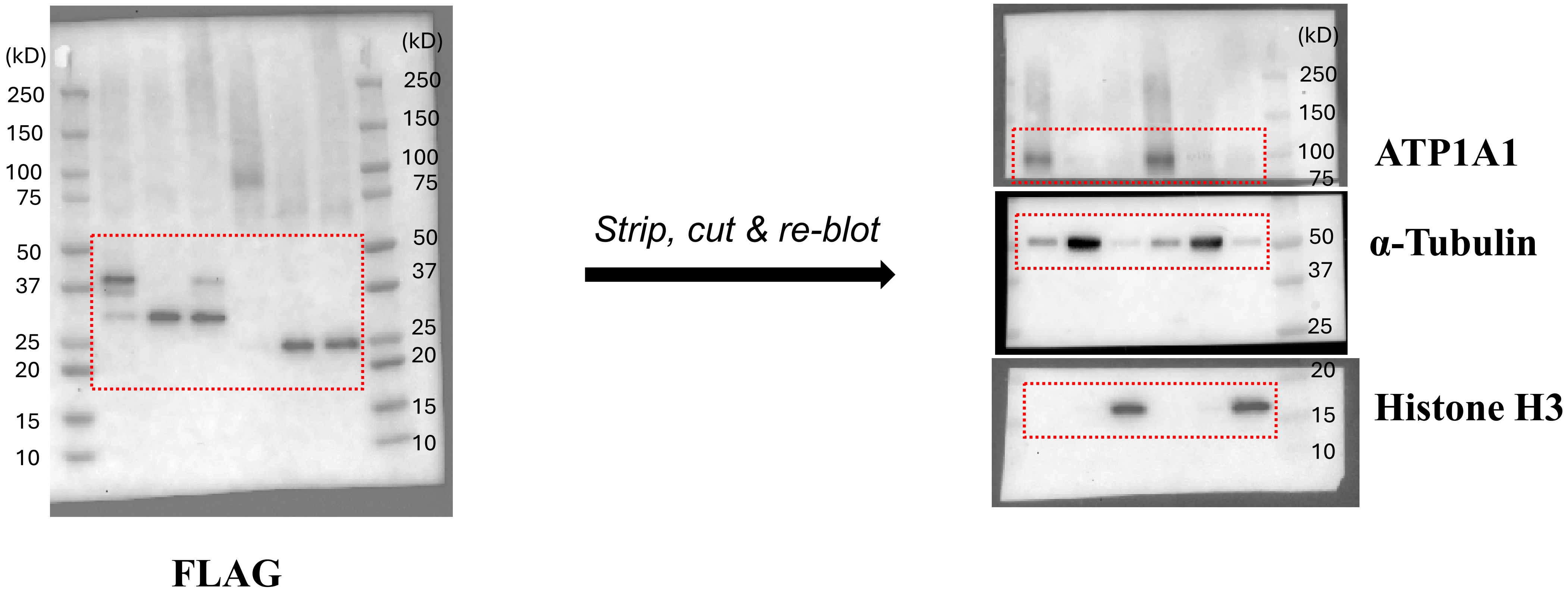

Full unedited blot for **Figure** 2E

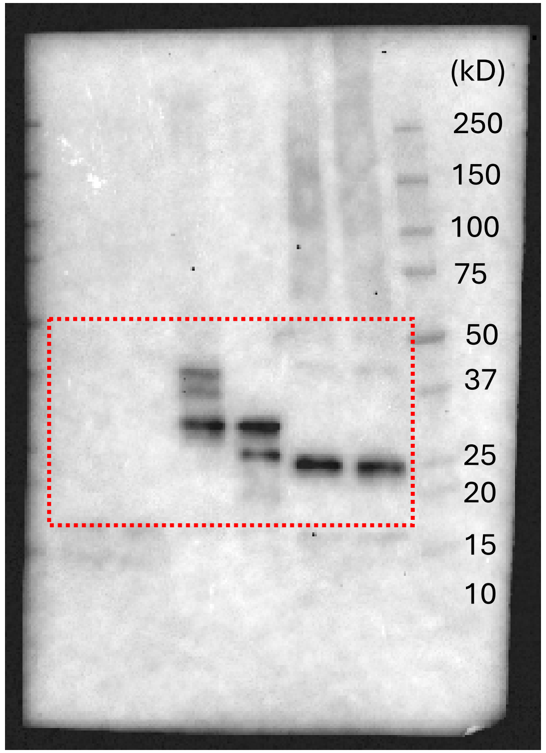

FLAG

Strip, cut & re-blot

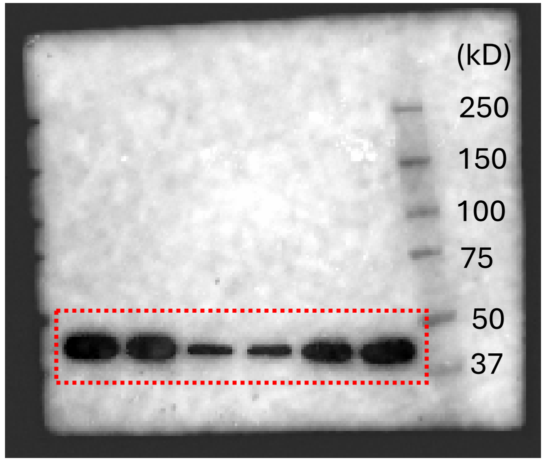

ACTB

Full unedited blot for **Figure 2F**

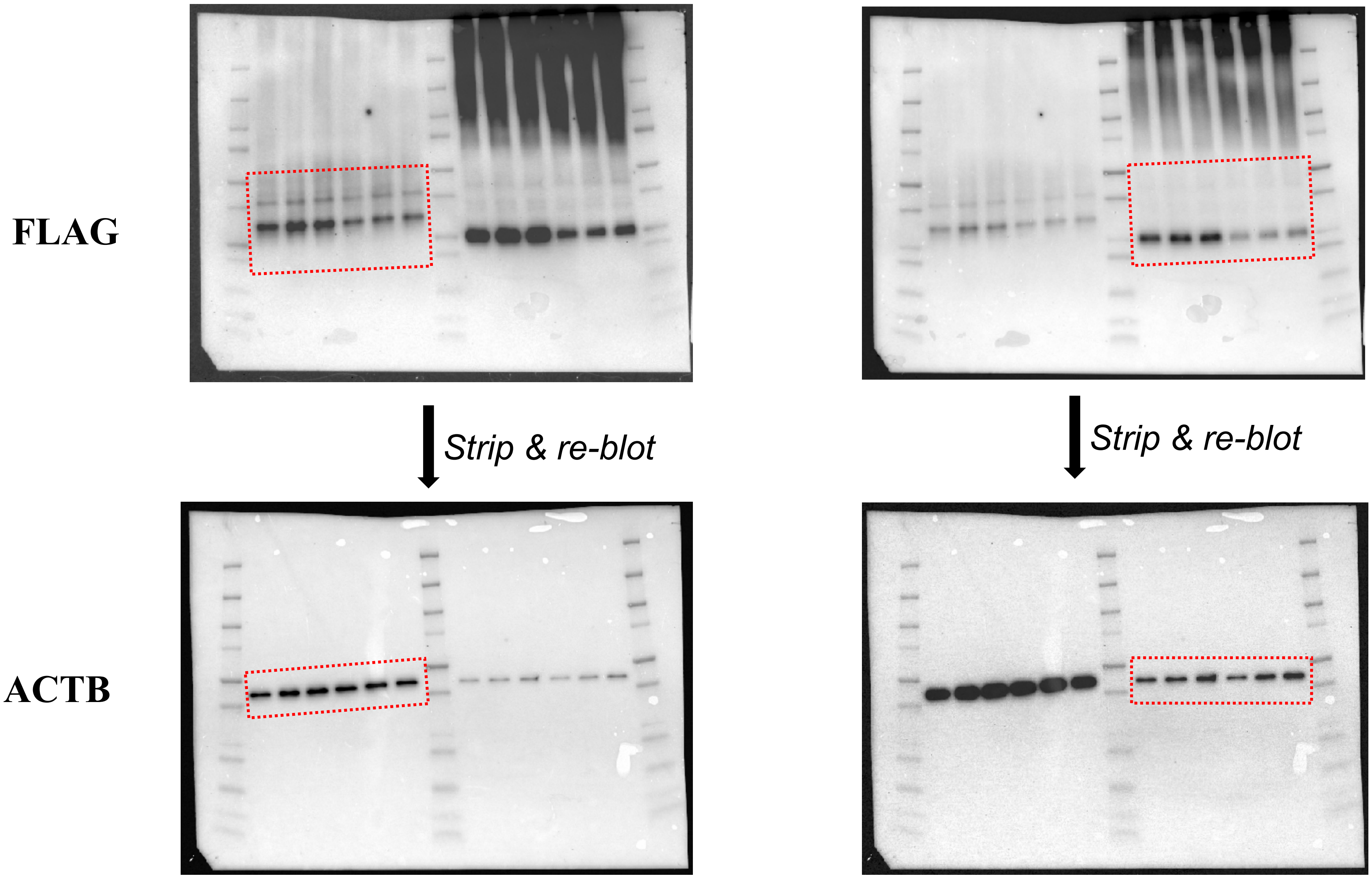

Full unedited blot for **Figure 2H**

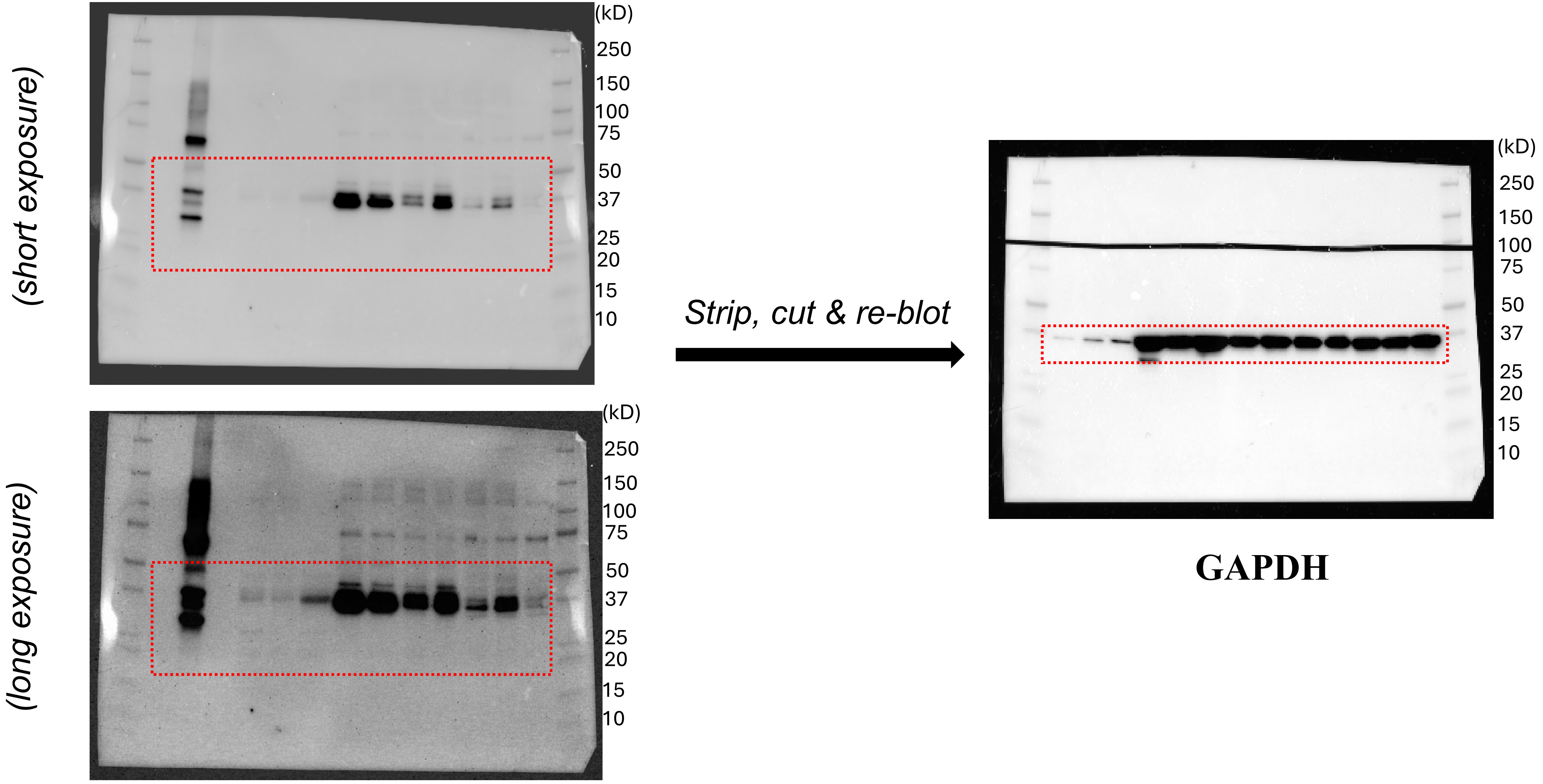

FNDC4 (Ab-4; OTI3E10)

Full unedited blot for **Figure 3C** (part 1)

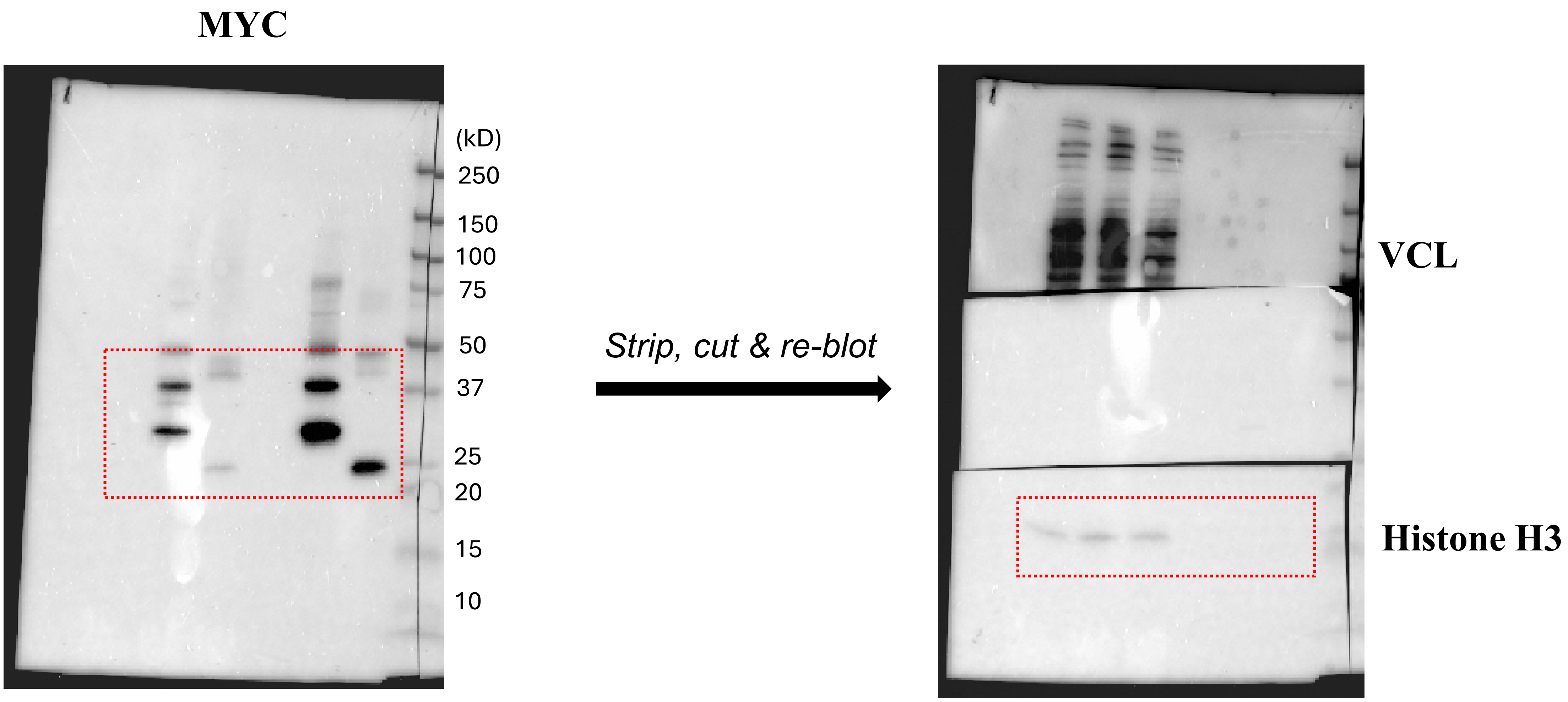

Full unedited blot for **Figure 3C** (part 2)

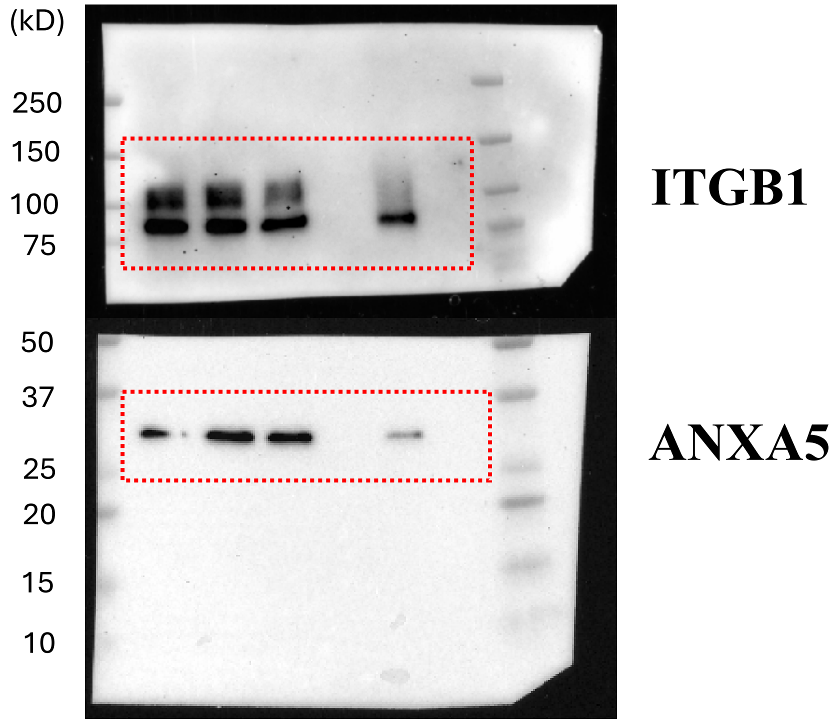

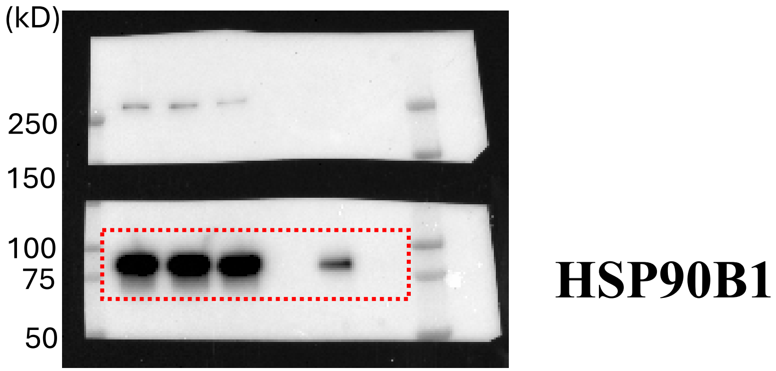

Full unedited blot for **Figure 6H**

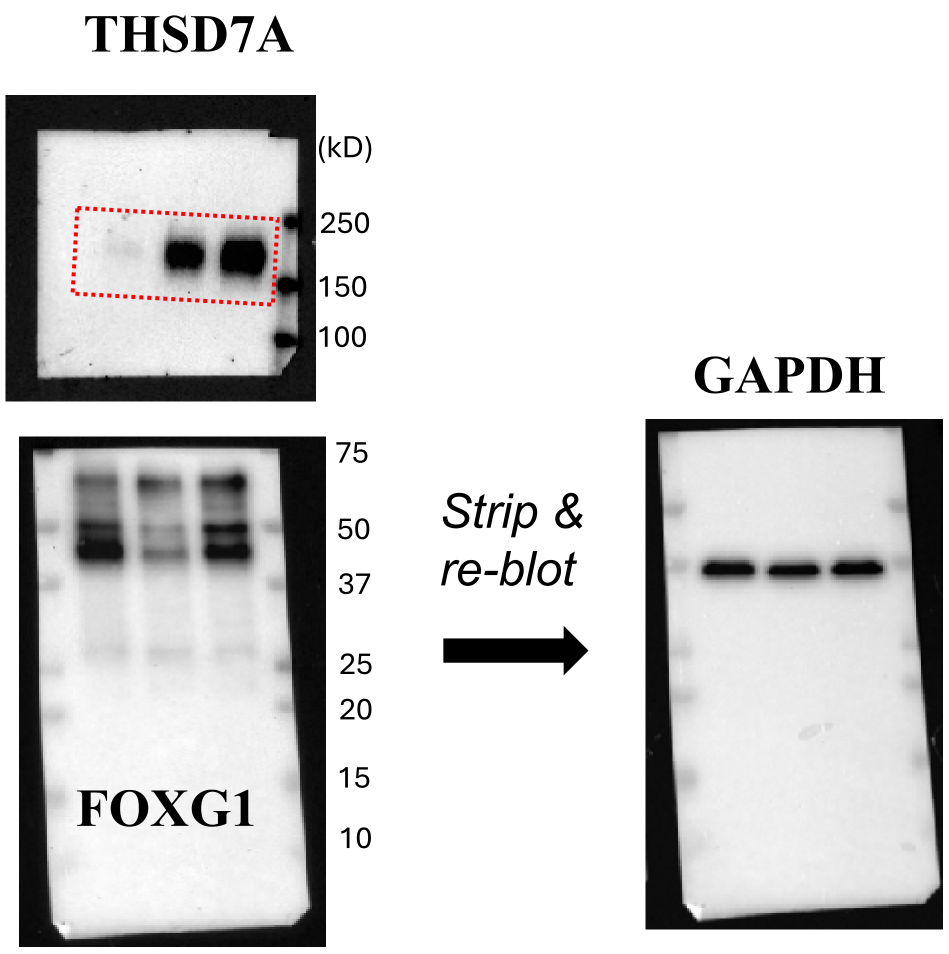

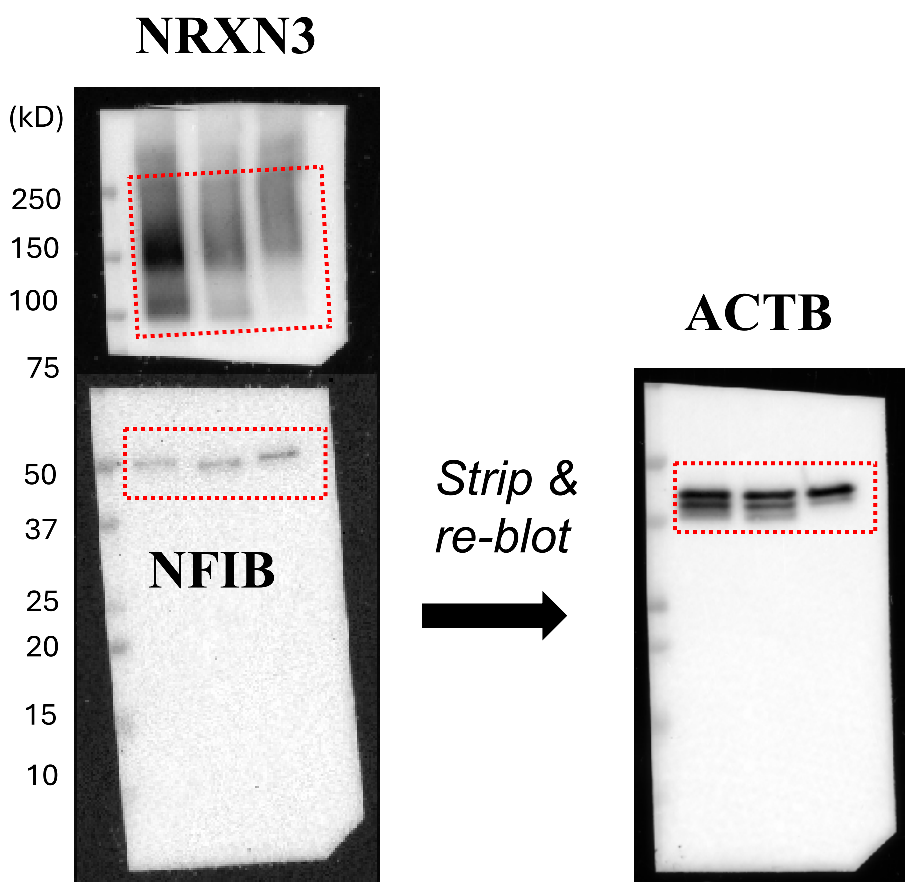

Full unedited blot for **Supplemental Figure S3D**

(short exposure)

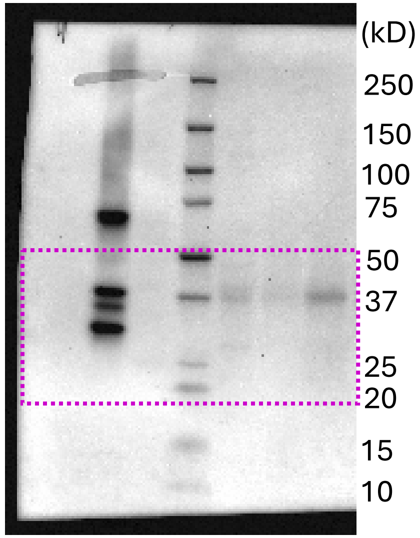

(long exposure)

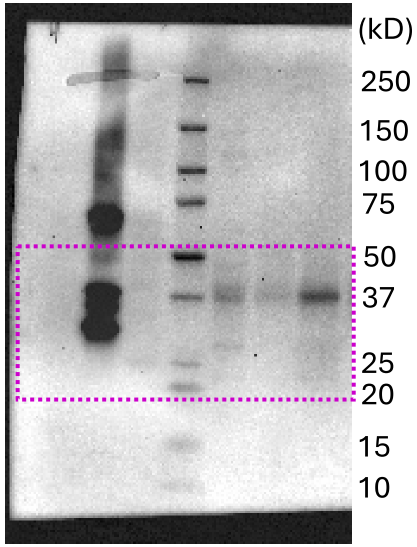

Strip, cut & re-blot

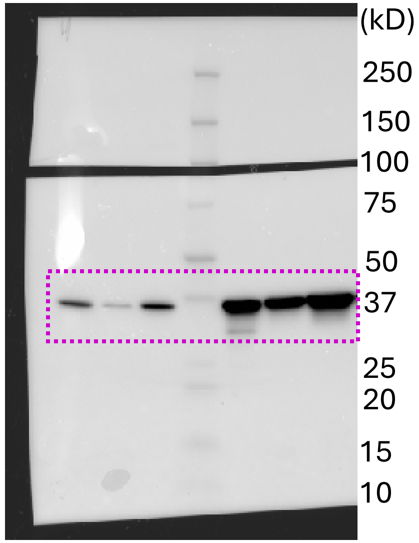

FNDC4 (Ab-4)

GAPDH

Blots in Supplementary *Fig.* ***S3E***

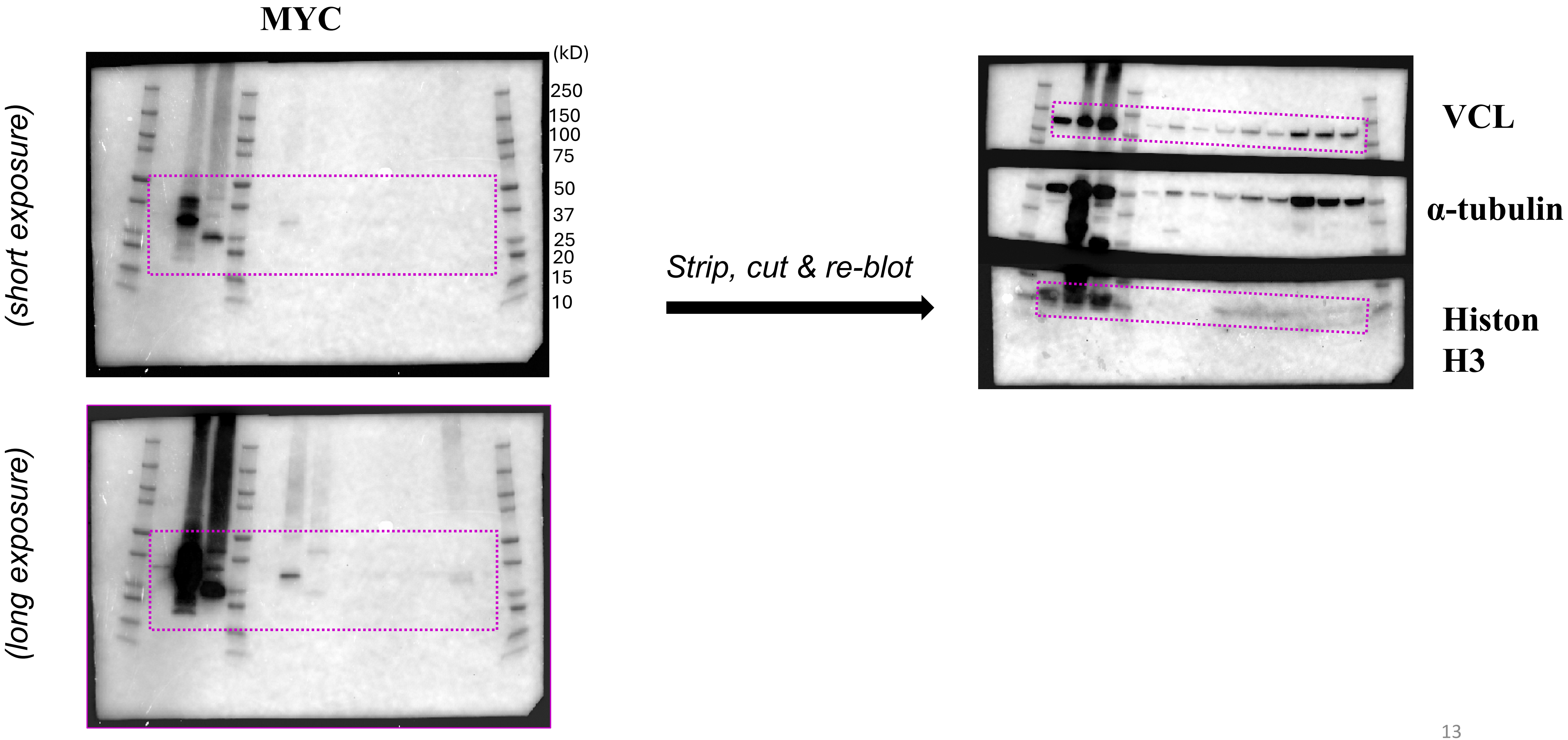

Supplement: Unedited blot and gel images [file jci-136-193204-s079.pdf]
